# Supplementary material for: Same-visit hepatitis C testing and treatment to accelerate cure among people who inject drugs (the QuickStart Study): a cluster randomised cross-over trial protocol
Source: BMJ Open. 2024 Jul 2;14(7):e083502. doi: 10.1136/bmjopen-2023-083502 (PMC11227801; doi:10.1136/bmjopen-2023-083502)
Supplement: Author's manuscript [file bmjopen-2023-083502.draft_revisions.pdf]

BMJ Open is committed to open peer review. As part of this commitment we make the peer review history of every article we publish publicly available.

When an article is published we post the peer reviewers' comments and the authors' responses online. We also post the versions of the paper that were used during peer review. These are the versions that the peer review comments apply to.

The versions of the paper that follow are the versions that were submitted during the peer review process. They are not the versions of record or the final published versions. They should not be cited or distributed as the published version of this manuscript.

BMJ Open is an open access journal and the full, final, typeset and author-corrected version of record of the manuscript is available on our site with no access controls, subscription charges or pay-per-view fees (http://bmjopen.bmj.com).

If you have any questions on BMJ Open's open peer review process please email info.bmjopen@bmj.com

BMJ Open

Same-visit hepatitis C testing and treatment to accelerate cure among people who inject drugs (The QuickStart Study): a cluster randomised crossover trial protocol

Journal:	*BMJ Open*
Manuscript ID	bmjopen-2023-083502
Article Type:	Protocol
Date Submitted by the Author:	22-Dec-2023
Complete List of Authors:	Doyle, Joseph S.; Monash University, Department of Infectious Diseases; Burnet Institute, Disease Elimination<br>Heath, Katherine; Burnet Institute<br>Elsum, Imogen; Burnet Institute<br>Douglass, Caitlin; Burnet Institute<br>Wade, Amanda; Burnet Institute<br>Kasza, Jessica; Monash University<br>Allardice, Kate; Burnet Institute, Disease Elimination<br>Von Bibra, Sally; Burnet Institute<br>Chan, Kico; Burnet Institute<br>Camesella, Beatriz; Burnet Institute<br>Guzman, Rodney; Burnet Institute<br>Bryant, Mellissa; Burnet Institute<br>Thompson, Alexander; St. Vincent's Hospital Melbourne<br>Stoové, Mark; Burnet Institute<br>Snelling, Thomas; Telethon Kids Institute, Wesfarmers Centre of Vaccines and Infectious Diseases; University of Sydney<br>Scott, Nick; Burnet Institute<br>Spelman, Tim ; Burnet Institute<br>Anderson, David; Burnet Institute<br>Richmond, Jacqui; Burnet Institute<br>Howell, Jess; Burnet Institute; St. Vincent's Hospital Melbourne<br>Andric, Nada; HepatitisWA<br>Dietze, Paul; Burnet Institute; Monash University<br>Higgs, Peter; Burnet Institute; La Trobe University, Public Health<br>Sacks-Davis, Rachel ; Burnet Institute<br>Forbes, Andrew; Monash University, Epidemiology and Preventive Medicine<br>Hellard, Margaret; Burnet Institute; Monash University, Epidemiology and Preventive Medicine<br>Pedrana, Alisa; Burnet Institute, Melbourne, Australia; Monash University, School of Public Health and Preventive Medicine
Keywords:	Hepatology < INTERNAL MEDICINE, Public health < INFECTIOUS DISEASES, INFECTIOUS DISEASES

TITLE:

Same-visit hepatitis C testing and treatment to accelerate cure among people who inject drugs (The QuickStart Study): a cluster randomised crossover trial protocol

AUTHORS:

Joseph Doyle*[1,2], Katherine Heath*[2], Imogen Elsum[2], Caitlin Douglass[2], Amanda Wade[2], Jessica Kasza[1], Kate Allardice[2], Sally Von Bibra[2], Kico Chan[2], Beatriz Camesella[2], Rodney Guzman[2], Mellissa Bryant[2], Alexander J Thompson[3], Mark Stoové[2], Tom Snelling[4,5], Nick Scott[2], Tim Spelman[2], David Anderson[2], Jacqui Richmond[2], Jess Howell[1,3], Nada Andric[6], Paul Dietze[1,2,7], Peter Higgs[2,8], Rachel Sacks-Davis[2], Andrew Forbes[1], Margaret Hellard[1,2], Alisa Pedrana[1,2]

*joint first author

INSTITUTIONAL AFFILIATIONS:

[1] Monash University, Melbourne, VIC, Australia

[2] Burnet Institute, Melbourne, VIC, Australia

[3] St Vincent's Hospital and the University of Melbourne, Melbourne, VIC, Australia

[4] Telethon Kids Institute, Perth, WA, Australia

[5] University of Sydney, Sydney, NSW, Australia

[6] Hepatitis WA, Perth, WA, Australia

For peer review only - http://bmjopen.bmj.com/site/about/guidelines.xhtml

[7] National Drug Research Institute, Curtin University, Melbourne, VIC, Australia

[8] La Trobe University, Melbourne, VIC, Australia

CORRESPONDING AUTHOR:

Prof Joseph Doyle

Professor of Infectious Diseases, Monash University, and Deputy Director, Disease Elimination Program, Burnet Institute

85 Commercial Road, Melbourne VIC 3004 Australia

Email: Joseph.doyle@monash.edu

Telephone: +61 3 9076 5436

ABSTRACT:

Introduction: Despite universal access to government funded Direct Acting Antivirals (DAAs) in 2016, the rate of hepatitis C treatment uptake in Australia has declined substantially. Most hepatitis C is related to injecting drug use; reducing the hepatitis C burden amongst people who inject drugs (PWID) is therefore paramount to reach hepatitis C elimination targets. Increasing DAA uptake by PWID is important for interrupting transmission and reducing incidence, as well as reducing morbidity and mortality and improving quality of life of people who inject drugs and meeting Australia's hepatitis C elimination targets.

For peer review only

For peer review only - http://bmjopen.bmj.com/site/about/guidelines.xhtml

Methods and analysis: A cluster randomised crossover trial will be conducted with three intervention arms and a control arm. Arm A will receive rapid hepatitis C virus (HCV) antibody testing; Arm B will receive rapid HCV antibody and rapid ribonucleic acid (RNA) testing; Arm C will receive rapid HCV antibody testing and same-day treatment initiation for HCV antibody positive participants; the Control Arm will receive standard of care. The primary outcomes will be (a) the proportion of participants with HCV commencing treatment and (b) the proportion of participants with HCV achieving cure. Analyses will be conducted on an intention-to-treat basis with mixed-effects logistic regression models.

Ethics and dissemination: The study has been approved by the Alfred Ethics Committee (Number HREC/64731/Alfred-2020-217547). Each participant will provide written informed consent. Reportable adverse events will be reported to the reviewing ethics committee. The findings will be presented at scientific conferences and published in peer-reviewed journals.

Registration details: ClinicalTrials.gov Identifier: NCT05016609

Trial progression: The study commenced recruitment on the 9 March 2022 and is expected to complete recruitment in December 2023.

For peer review only - http://bmjopen.bmj.com/site/about/guidelines.xhtml

STRENGTHS AND LIMITATIONS OF THIS STUDY:

- The QuickStart Trial will recruit from a focused population, people with current or past history of injecting drugs, who are most at risk of hepatitis C.
- A cross-over clustered randomised control trial design will be used to increase study power by leveraging within-site comparisons.
- Four study conditions will be investigated to analyse three rapid point-of-care (POC) testing and treatment interventions, compared with a control arm. One arm investigates a rapid POC antibody test and same day treatment - an intervention that could be utilised in resource limited settings if found to be effective.
- Medical record review will be used in addition to in-person participant follow-up to monitor study outcomes and reduce loss-to-follow up.
- The participant recruitment strategy counteracts several biases present in observational studies, but participation is voluntary, so a risk of selection bias remains.

For peer review only

For peer review only - http://bmjopen.bmj.com/site/about/guidelines.xhtml

1. INTRODUCTION

As of 2019, approximately 58 million people worldwide were living with hepatitis C (HCV), causing around 290,000 deaths annually [1]. In Australia, approximately 118,000 people were living with HCV at the end of 2020 [2].

Direct-acting antiviral medications (DAAs) are highly effective (cure rates >90%), have minimal side-effects and require only 8-12 weeks of daily tablets. The Australia Pharmaceutical Benefits Scheme is a government program that subsidises medicines to improve affordability [3]. In 2016, the Pharmaceutical Benefits Scheme negotiated a fixed-price contract providing access to DAAs for every Australian living with hepatitis C, giving Australia a unique opportunity to eliminate HCV [4,5].

Most untreated HCV infections in Australia are among people with a history of injecting drug use [2,6]. Models of HCV epidemics where transmission is driven by injecting drug use suggest that treating people who inject drugs (PWID) and people with a history of injecting drugs will lead to substantial reductions in HCV incidence and liver-related morbidity and mortality [7–9]. Indeed, models have shown that treating as few as 6% of the estimated population of PWID in Australia annually for ten years would reduce new HCV infections by 80% and HCV

For peer review only - http://bmjopen.bmj.com/site/about/guidelines.xhtml

87 prevalence in PWID by 10% [10,11]. However, in Australia, PWID remain under-diagnosed, under-treated and under-serviced in HCV care and healthcare more broadly [2,12–14].

89 Unrestricted access to DAAs following government subsidy in 2016 resulted in an initial spike in treatment uptake due to increased availability of the medication to those who had previously been unable to access it. However treatment numbers have since declined, exacerbated by the COVID-19 pandemic [2,15,16]. There exist service, provider and individual barriers to accessing HCV care [17]. One service-related barrier among PWID is the current need for multiple venepunctures in order to be diagnosed with HCV in Australia, particularly as venous access can be challenging in PWID [18,19]. Current clinical guidelines and funding rules require specific assessments before treatment can be started [20] necessitating multiple clinic visits. Data from the Australian Needle and Syringe Program Survey estimates that among the 93,000 PWID in Australia, the majority (89%) had a lifetime history of HCV antibody testing [21]. However, individuals are gradually lost throughout the cascade of care. Of Australian Needle and Syringe Program Survey participants reporting testing positive to an HCV antibody test, only 32% reported accessing HCV treatment up to 2020 (prior to the COVID19 pandemic) [22].

For peer review only - http://bmjopen.bmj.com/site/about/guidelines.xhtml

Point-of-care (POC) HCV testing can simplify diagnosis of HCV in healthcare settings. The emergence of rapid POC testing for both HCV antibodies and RNA has made this feasible. The OraQuick® rapid HCV antibody test is self-contained and uses fingerstick capillary blood samples to provide results in around 20 minutes. The GeneXpert ® HCV Viral Load test detects HCV RNA using fingerstick capillary blood to provide results in around 60 minutes. These tests have been shown to be highly sensitive and specific, [23,24] and overcome many of the barriers to treatment associated with the conventional care model.

Studies suggest POC testing is acceptable among PWID and can be feasibly integrated into health services [19,25–27]. However, limited data have assessed the impact of POC testing on treatment uptake. One study in France found a larger proportion of individuals who received a POC test completed HCV screening and were linked to care compared to those with standard serological testing [28]. Provisional data in Australia found POC testing through needle and syringe exchange programs reduced time to treatment [29]. In Connecticut, patients selecting POC testing were more likely to be linked to HCV treatment than those opting for standard laboratory testing [30]. These studies suggest that POC testing has a positive effect upon retention in HCV care [27,29]. Beyond rapid testing, the feasibility of same-day testing and treatment with DAAs has been demonstrated: preliminary studies have shown high levels of

For peer review only

linkage to care following same-day testing and treatment [31]. Same-day testing and treatment has also been shown to achieve high cure rates compared to facilitated referral [32]. However, only a limited number of studies that have assessed the impact on retention in care of same-day treatment following POC testing for HCV [31].

From these existing data, there are broadly three rapid testing models of care being contemplated and deployed in real-world settings: firstly, POC antibody testing alone (corresponding to Arm A of QuickStart Trial, more fully defined below); secondly, combined POC antibody and RNA testing (Arm B); and thirdly, combined POC testing and same-day treatment (Arm C). While promising in pilot studies, none of these models have been tested formally in clinical trials. Clinical trial evidence is required to determine effectiveness of POC testing with or without treatment compared with the current standard of clinical care.

The QuickStart Trial will firstly determine the effectiveness of rapid POC testing in primary healthcare settings upon treatment uptake. We hypothesise that rapid antibody testing alone (Arm A), and rapid antibody followed by immediate RNA testing (Arm B), will increase treatment uptake compared with standard clinical care (the control arm). Secondly, QuickStart will determine the effectiveness of POC antibody testing followed by same-day treatment (Arm C) upon cure. We hypothesis that testing and same-day treatment with increase cure compared

For peer review only

137 with standard clinical care. Our secondary outcomes will also compare each of the three

138 intervention models to each other for any incremental benefit on treatment uptake and cure.

139

For peer review only

For peer review only - http://bmjopen.bmj.com/site/about/guidelines.xhtml

2. METHODS AND ANALYSIS

2.1 Trial design

A clustered randomised crossover trial with six treatment sequences will evaluate the effects of rapid POC testing on retention of participants with HCV care and achievement of cure. Each site will have a standard of care control period randomly occurring before or after one of three intervention arms, allowing for intra-site comparison. Each site will have a standard of care control period randomly occurring before or after one the three intervention arms, allowing for intra-site and inter-site comparisons to be combined in the estimation of treatment effects. The cross-over design has advantages in ensuring that any external changes that occur over time can be accounted for, which a simple before and after intervention comparison would not allow. External changes might occur at the clinic level, such as services and staff becoming more aware or skilled in offering hepatitis C testing, or they may occur at a system level, such as changes in access to testing, care or treatment. There will be a one-month washout period between arms at each study site. Participant recruitment began in March 2022 and is intended to continue for up to 24 months. We engaged the University of New South Wales to convene a Community Reference Panel to provide input and feedback into the design of the research and study procedures. The panel involved ten members from the study target audience who were involved in two separate consultations in 2021.

For peer review only - http://bmjopen.bmj.com/site/about/guidelines.xhtml

2.2 Study setting

The trial will be conducted at primary healthcare services in metropolitan and rural settings in Australia with:

- high caseloads of people who currently or previously injected drugs and are at risk of HCV infection;
- a General Practitioner and/or a Nurse Practitioner experienced in testing and treating HCV

2.3 Recruitment

Eligible clinics will be identified through consultation with government, community, clinical and research networks in viral hepatitis in Australia. Within clinics, potentially eligible participants will be approached by a clinician, nurse or community health worker. Participant-facing study promotion including posters and leaflets will be used at the discretion of participating clinics.

For peer review only

2.4 Participant eligibility

Study inclusion criteria for participants are as follows:

- current or former injecting drug use (i.e., injected drugs at least once)
- aged 18 years or over
- attending a participating primary healthcare for any reason
- no previous treatment with DAAs
- medicare eligible
- able to speak and understand English

Exclusion criteria are as follows:

- women known to be currently pregnant or who are breastfeeding
- individuals currently engaged in HCV treatment
- unable to provide informed consent
- tested for HCV in the past 3 months
- previous successful treatment with interferon-based therapy for HCV

For peer review only

For peer review only - http://bmjopen.bmj.com/site/about/guidelines.xhtml

2.5 Study arms

This study will implement three intervention arms and a control arm, as shown in Figure 1. Final recruitment, retention and outcome assessments will be presented in accordance with the CONSORT statement, including CONSORT diagrams. Arm A will implement a rapid POC antibody test in addition to standard of care (SOC) confirmatory RNA testing and bloods tests at the baseline visit. Arm B will implement a rapid POC antibody and POC RNA test in addition to SOC bloods tests at the baseline visit. Arm C will implement a rapid POC antibody test and same-day treatment with DAAs for antibody positive participants in addition to standard bloods tests. The control arm will implement the SOC in operation at each clinic.

All participants will be administered a clinical and behavioural questionnaire at their baseline visit. All arms are outlined in detail below.

2.5.1 <u>Arm A: Rapid POC antibody + SOC</u>

Baseline visit:

- Participants will receive an OraQuick® rapid HCV antibody test using a fingerstick specimen, according to the manufacturer's instructions, with the result documented at

For peer review only

20 minutes. The result will be provided to the participant as a preliminary result with laboratory testing on a venous sample required to confirm the POC test result. If the POC result is positive, blood samples will be collected for any confirmatory laboratory antibody and RNA testing and SOC pre-treatment screening. If the POC result is negative, confirmatory testing will be offered but the participant is given the option to decline.

Week 0-2 (confirmatory lab results):

- If the confirmatory laboratory HCV Antibody result is **negative**, the nurse will communicate this to the participant. The participant will complete the study here. Standard harm minimisation messaging and window periods for detection of HCV will be discussed with the participant.
- If the confirmatory laboratory HCV antibody result is **positive** and the RNA result is **negative**, the nurse will contact the participant by phone to inform them of their result - prior exposure to HCV, no current infection. The participant will complete the study here.
- If the confirmatory laboratory HCV antibody result is **positive** and the RNA result is **positive**, the nurse will contact the participant by phone to inform them of their result

For peer review only

For peer review only - http://bmjopen.bmj.com/site/about/guidelines.xhtml

of current HCV infection and arrange a follow up appointment to discuss treatment as per the clinic usual standard of care.

Week 2-12 (HCV RNA positive):

- HCV RNA positive participants will be followed-up as per usual clinic SOC.

Week 12 (HCV RNA positive):

- The study nurse will review the medical records of consented participants to determine whether participants with HCV were provided with a DAA prescription within 12 weeks of enrolment in the study.
- All participants who have been provided with a DAA prescription will be contacted by the study nurse to ask whether they started treatment and if so, when.

12 weeks following treatment completion (Sustained Virological Response (SVR) or cure assessment):

- The medical records of all participants confirmed through medical record review to have started DAA treatment will be reviewed to determine if they have had a SVR blood test anytime from 4 -12 weeks following their treatment completion date [33]. Reinfection will be differentiated from treatment failure due to relapse by assessing (1)

For peer review only - http://bmjopen.bmj.com/site/about/guidelines.xhtml

genotype or subtype, and (2) viral sequencing at a reference laboratory, whenever possible.

- Participants who have not had a SVR visit, will be contacted by the study nurse approximately 12 weeks following the scheduled treatment completion date (determined by the date they received their script and duration of treatment) to request that they attend an SVR visit.

2.5.2 Arm B: Rapid POC antibody + rapid POC RNA + SOC

Baseline visit:

- Participants will receive an OraQuick® rapid HCV antibody test using a fingerstick specimen, according to the manufacturer's instructions, with the result documented at 20 minutes. The result from the 20-minute time point will be provided to the participant as a preliminary result, with laboratory testing on a venous sample required to confirm. Blood samples will be collected for any confirmatory laboratory testing and standard of care pre-treatment screening.

For peer review only

For peer review only - http://bmjopen.bmj.com/site/about/guidelines.xhtml

- Participants with a **negative** OraQuick® result will be provided with this preliminary result and informed that they will be contacted within the next two weeks with any laboratory test results.
- Participants with a **positive** OraQuick® result will be offered POC RNA testing using the Cepheid GeneXpert™ HCV RNA fingerstick test. The study nurse will conduct POC RNA testing using a fingerstick blood sample according to the manufacturer's instructions, with results in ~60 minutes. The study nurse will determine with the participant how they would like to receive these results – via remaining on site or by phone call. If the HCV POC RNA is **negative** the study nurse will inform the participant of their result, either in person or by telephone, and they will be contacted within the next 2 weeks with confirmatory laboratory test results.
- If the HCV POC RNA is **positive**, the study nurse will inform the participant of their result either in person or by telephone and they will be contacted within the next two weeks with confirmatory laboratory test results.

Week 0-2 (confirmatory lab results):

- If the confirmatory laboratory RNA results are **negative** the nurse will inform the participant and the participant will complete the study. Standard harm minimisation

For peer review only

For peer review only - http://bmjopen.bmj.com/site/about/guidelines.xhtml

messaging and window periods for detection of HCV will be discussed with the participant.

- If the laboratory HCV RNA is **positive** the study nurse will contact the participant to provide their results of current HCV infection and discuss follow up and treatment according to the practice of the clinic.

Week 2-12 (HCV RNA positive):

- Participants with HCV will be followed-up as per usual clinic SOC.

Week 12 (HCV RNA positive):

- The study nurse will review the medical records of consented participants to determine whether participants with HCV were provided with a DAA prescription within 12 weeks of enrolment in the study.
- All participants who have been provided with a DAA prescription, will be contacted by the study nurse to ask whether they started treatment and if so, when.

12 weeks following treatment completion (SVR):

SVR will be assessed as previously described for Arm A.

For peer review only - http://bmjopen.bmj.com/site/about/guidelines.xhtml

For peer review only

2.5.3 Arm C: Same-visit rapid POC antibody + treatment commencement

Baseline visit:

- Participants will receive an OraQuick® rapid HCV antibody test using a fingerstick specimen, according to the manufacturer's instructions, with the result documented at 20 minutes. The result from the 20-minute time point will be provided to the participant as a preliminary result, with laboratory testing on a venous sample required to confirm. Blood samples will be collected for any confirmatory laboratory testing and standard of care pre-treatment screening.
- Participants with a **negative** OraQuick® result will be advised that the POC test result will be confirmed by standard pathology results and they will be contacted in 1-2 weeks with these results.
- Participants with a **positive** OraQuick® result will be provided with two weeks of Sofosbuvir/Velpatasvir (Epclusa) and will be instructed to begin treatment. In this sense, Arm C uses presumptive treatment, where 14 days of treatment will be dispensed prior to laboratory confirmed diagnosis [34]. Blood samples will be sent for confirmatory HCV antibody and RNA testing. Participants of childbearing potential will be screened for possible pregnancy and if indicated, a urinary pregnancy test will

For peer review only

be performed before dispensing Epclusa. If the pregnancy test is positive, they will not be provided with study drug and will be referred to standard of care. Medical records will be reviewed for potential drug-drug interactions with Epclusa, and participants will be asked about medications they are currently taking. Participants taking medications with red or orange drug-drug interactions will not be provided with study drug and will be referred to standard of care.

Week 1-2 (Confirmatory Results)

- Participants will be contacted with the results of their confirmatory laboratory blood tests. Participants with a **positive** confirmatory antibody and **negative** RNA test result will be contacted with this result of prior exposure, no current infection and instructed to discontinue treatment and dispose of any unused medications. They will complete the study here. Standard harm minimisation messaging and window periods for detection of HCV will be discussed with the participant.
- Participants with a **positive** confirmatory antibody and **positive** confirmatory RNA result will be contacted to arrange on-going treatment through the study, a further four weeks of sofosbuvir/velpatasvir will be dispensed. Further assessment maybe required based on the laboratory blood test results:

For peer review only

For peer review only - http://bmjopen.bmj.com/site/about/guidelines.xhtml

- If the participant received an AST:Platelet Ratio Index score of < 1.0, they will be instructed to continue treatment as they were originally instructed.
- If the participant received an AST:Platelet Ratio Index score of ≥1.0, they will be instructed to continue taking the DAAs and will be referred to a specialised.
- Participants found to have renal impairment (eGFR<30 ml/min) or coinfected with chronic hepatitis B will be instructed to cease treatment and will be referred to a specialist.
- Participants found to have Human Immunodeficiency Virus infection will be instructed to continue the DAAs and be referred to a specialist.

Following specialist assessment, the participant may be required to change medications or alter their treatment course, according to the specialist's recommendations.

Week 6 (HCV RNA positive):

- Participants on treatment will be asked to attend a follow-up appointment where they will be provided with a further four weeks of sofosbuvir/velpatasvir.

Week 10 (HCV RNA positive):

For peer review only - http://bmjopen.bmj.com/site/about/guidelines.xhtml

- Participants who have received 10 weeks of treatment will be contacted to arrange a follow up appointment to receive the final two weeks of treatment.

12 weeks following treatment completion (SVR):

SVR will be assessed as previously described for Arm A.

2.5.4 Control arm: standard of care testing and treatment

All participants will be tested, treated and followed up according to the usual practice of the clinic for delivery of test results and ongoing HCV treatment. The participants medical record will be reviewed at the study close to assess testing and treatment uptake.

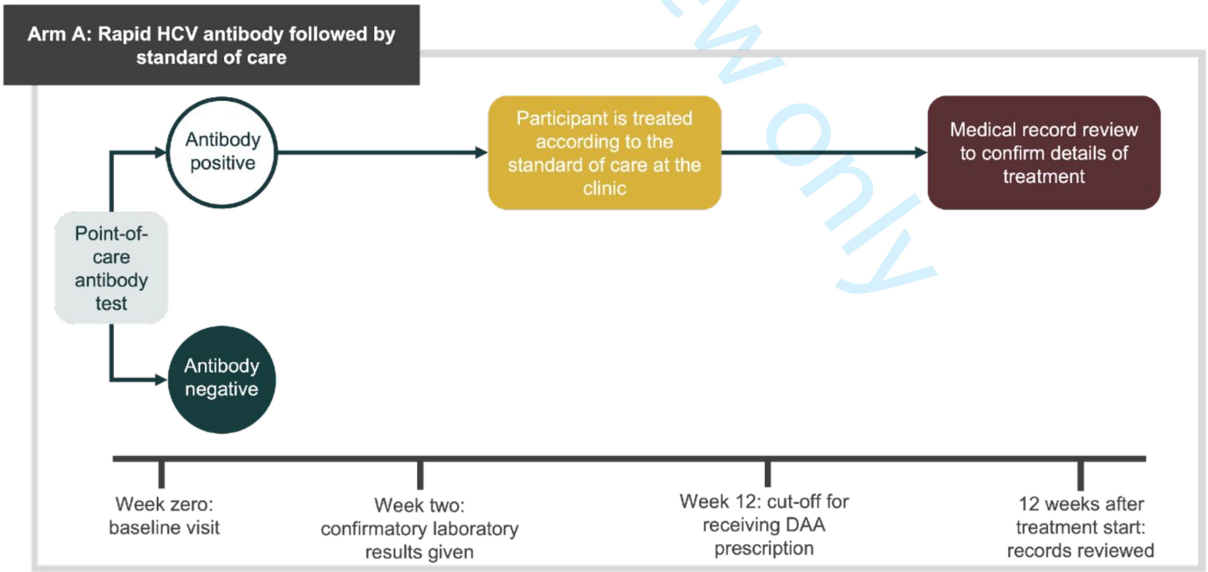

Figure 1A. Sequence of events in Arm A: rapid HCV antibody testing followed by standard of care.

For peer review only

For peer review only - http://bmjopen.bmj.com/site/about/guidelines.xhtml

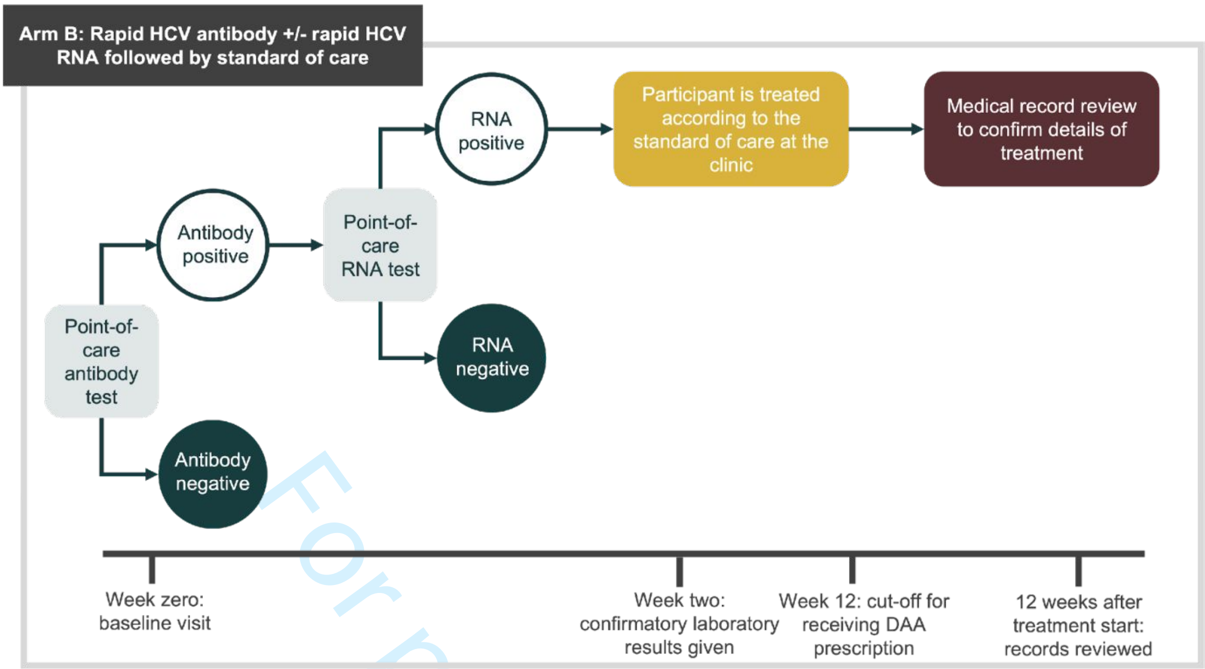

Figure 1B. Sequence of events in Arm B: rapid HCV antibody +/- rapid HCV RNA followed by standard of care.

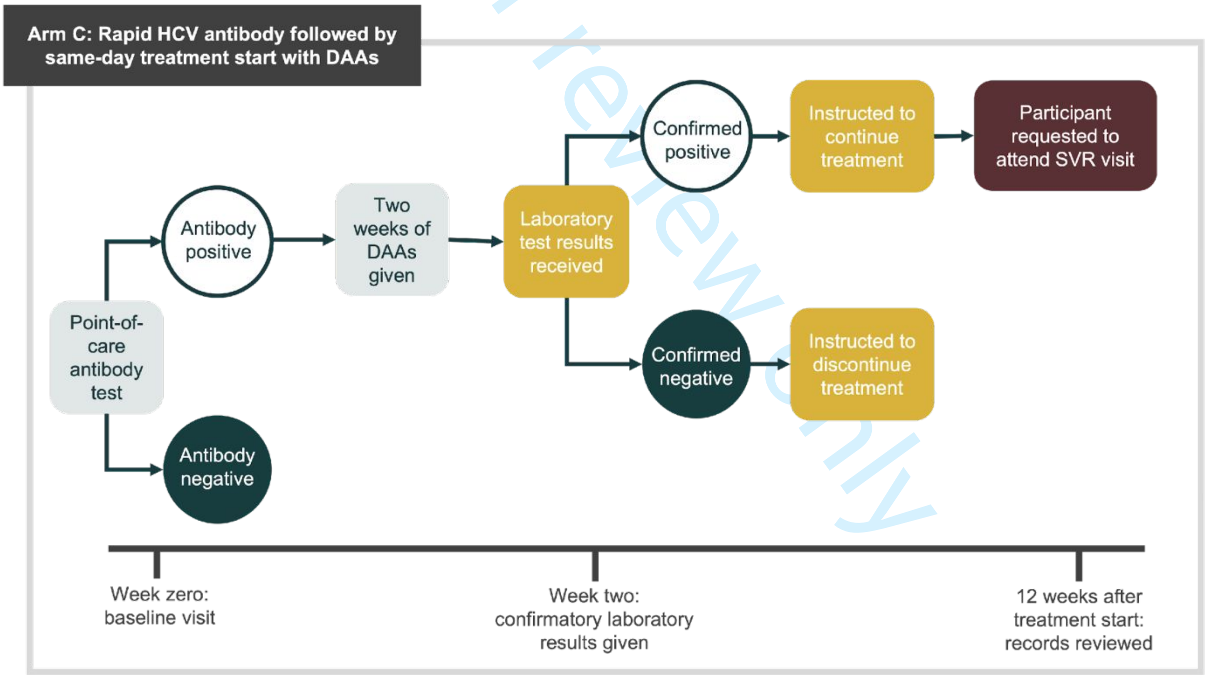

Figure 1C. Sequence of events in Arm C: rapid HCV antibody followed by same-day treatment with DAAs.

For peer review only - http://bmjopen.bmj.com/site/about/guidelines.xhtml

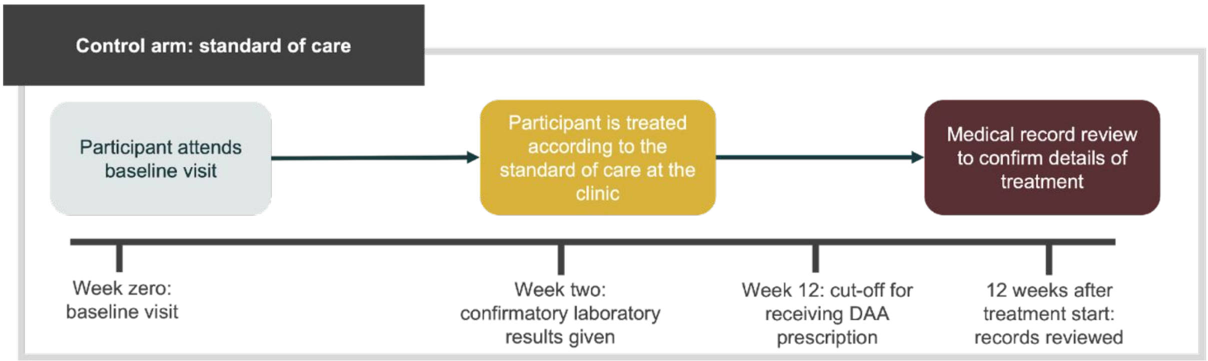

Figure 1D. Sequence of events in the Control arm: standard of care

2.6 Study medication

For Arms A, B and the Control, medications will be funded by the PBS and dispensed utilising standard pharmacy practices. The type of DAAs used to treat participants with HCV in these Arms will be at the discretion of the clinical service where the participant is enrolled. For Arm C, study medications will be stored and dispensed by participating study sites. The medication used in Arm C of this study is a 12-week course of oral combination therapy sofosbuvir/velpatasvir (Epclusa™).

Epclusa tablets contain 400mg of sofosbuvir and 100mg of velpatasvir, to be taken orally once daily. Epclusa is TGA approved with ARTG ID 266823 [35].

For peer review only

For peer review only - http://bmjopen.bmj.com/site/about/guidelines.xhtml

2.7 Assignment of interventions

Intervention arms will be assigned to sites using random number generation in a centralised system. Sites will be randomised in batches of six to ensure a balance of the number of sites in each arm over time. Sites will be informed of their allocation to each of the two consecutive arms separately. Due to the nature of the study design, it is not possible to blind clinics or participants during all phases of the intervention. All sites randomised to Arms A, B or C in the first period will know that they will be allocated to the Control Arm after the cross-over period, due to the nature of the study design. Sites allocated to the Control Arm in the first period will be blinded as to which Arm they will be randomised to, following the cross-over washout period (see Figure 1).

For peer review only

2.8 Study outcomes

The two primary outcomes of this trial include:

(1) The proportion of participants commencing HCV treatment in each arm. Commencement of HCV treatment is defined as receipt of a script for treatment in arms A, B and Control and

For peer review only - http://bmjopen.bmj.com/site/about/guidelines.xhtml

as commencement of treatment with study drug in Arm C. The proportion commencing treatment in each intervention arm will be compared to the Control arm.

(2) The proportion of participants with HCV who start treatment achieving cure. Cure will be assessed by review of medical records for a clinic visit to assess SVR; if no visit has taken place, the study nurse will contact the participant to arrange one in all Arms except the Control.

Commencing treatment is an important and easily measurable outcome in clinical practice. The rationale for two primary outcomes is that Arm C itself involves providing treatment to everyone found to have HCV as part of its intervention.

Secondary outcomes of this study include:

(1) Time to treatment commencement, defined as number of days from first test to first dose of treatment.

(2) Case finding, defined as the proportion of people diagnosed with HCV among all those recruited

(3) Cost-effectiveness, defined as the incremental cost to find and commence treatment among one person living with HCV from a health system perspective

For peer review only

(4) Participant acceptability, based on qualitative evaluation subject to further sub-study design and ethical approval

2.9 Sample size

Comparisons of primary interest are comparing outcome 1 (Treatment commencement) in Control and Arm A; outcome 1 in Control and Arm B; and outcome 2 (HCV cure) in Control and Arm C. We consider sample size calculations with a minimum of 80% power and a significance level of 0.05/3 = 0.0167. Comparisons of Control vs A are expected to result in the smallest required sample sizes, so sample size calculations focus on this comparison. We use the Shiny CRT app [36] for sample size calculations, assuming that the t- distribution is used instead of the normal approximation (accounting for small numbers of clusters) [37].

We suppose that five sites are assigned to each of the six treatment sequences. We found that with 24 participants recruited in each cluster in each period, power will be greater than 80% to detect a change in proportions of participants starting HCV treatment of 30% to 50% where ten clusters are allocated to Arm A. Due to greater portion of participants expected to start treatment in Arm B (70%) and Arm C (90%), power >80% is achieved with eight clusters assigned to the sequences that involve Arms B or C (i.e. with 4 clusters randomised to each of

For peer review only - http://bmjopen.bmj.com/site/about/guidelines.xhtml

the Arm B then Control; Control then Arm B; Arm C then Control; and Control then Arm C sequences). The proportion of participants expected to achieve cure in each arm are 10% among controls, 30% in Arm A, 50% in Arm B, and 70% in Arm C. Allowing for 20% loss to follow up of sites and of participants, 30 participants per site per period would need to be recruited. Given these results, we will instruct sites to aim for 40 participants per site per period to counteract instances where some sites are unable to achieve 30 participants per site per period; some sites achieving 40 participants will balance sites which achieve <30. This would imply a maximum total of 2,400 participants would need to be recruited.

2.10 Statistical analysis

Results from the trial will be reported according to the CONSORT extension statement for cluster randomised trials [38], or a CONSORT extension statement for cluster randomised crossover trials if one is available prior to end of data collection. Clinic and patient demographics will be described by site and study period. Analyses will be conducted on an intention-to-treat basis, with patients analysed according to the randomised treatment of their cluster at the time of their inclusion in the study. All available binary outcomes will be analysed at the patient level using mixed-effects logistic regression models with random intercepts for

For peer review only

431 clinic and clinic-period and fixed effects for interventions [39]. Odds ratios and 95%
432 confidence intervals will be reported; in addition, we will calculate and report risk differences
433 and 95% confidence intervals.

434 There are three main comparisons proposed, namely, treatment uptake outcome in Arm A
435 versus control (comparison one) and Arm B versus control (comparison two), and HCV cure
436 in Arm C versus control (comparison three). Incremental benefits of Arm B to Arm A, and
437 Arm C to Arm B, and Arm C to Arm A on treatment uptake and HCV cure will be performed
438 in sub-group analyses. Data from controls in each arm will be pooled and a single model fitted
439 for each outcome.

440 Randomisation will occur in batches of six, performed by the coordinating centre, and
441 concealed from investigators and sites until after site governance approcal. Since
442 randomisation is occurring within batches of six services, fixed effects for batch will be
443 included in these models. The Kenward-Roger correction will be applied to adjust for the small
444 number of clinics [40]. Estimates of intra-cluster correlations (within-period and between-
445 period) will be reported. Time-to-event outcomes such as time to complete diagnosis will be
446 analysed using Cox proportional hazards models with shared-frailty terms for site and fixed
447 terms for treatment group and randomisation batch. There may be imbalances between sites

For peer review only

with respect to various clinic and patient-level characteristics, and secondary analyses will adjust for these.

Sensitivity analyses to investigate the impact of different assumptions about missing data on study conclusions will be performed. Initially, two scenarios will be considered: "best-worst" and "worst-best". In the "best-worst" scenario, all patients with missing data in clinics during periods of administration of Arms A, B or C will be assumed to have commenced HCV treatment, and all patients in control periods will be assumed not to have commenced HCV treatment. In the "worst-best" scenario, all patients with missing data in clinics during periods of administration of Arms A, B or C will be assumed to not have commenced HCV treatment, and all patients in control periods will be assumed to have commenced HCV treatment. Similar scenarios for the SVR outcome will be considered. Should these analyses lead to different study conclusions, further analyses including terms predictive of missingness will be conducted or using multiply imputed datasets will be considered.

For peer review only

For peer review only - http://bmjopen.bmj.com/site/about/guidelines.xhtml

3 ETHICS AND DISSEMINATION

3.1 Ethical review

This study has been approved by the Alfred Ethics Committee (Number: HREC/64731/Alfred-2020-217547). Informed consent will be obtained from all participants prior to enrolment. Potential participants will be presented with detailed information about the study procedures, objectives, risks and benefits. Participants will be able to refuse or terminate participation at any time. Participants will be reimbursed $40 at their baseline visit and $80 for their SVR visit. Reimbursement will be by Coles/Myer voucher or by direct funds bank transfer (EFT/PayID).

3.2 Data monitoring

The trial will be monitored by an independent Data Safety and Monitoring Board with an independent statistician. There will be pre-defined periodic analysis to assess safety endpoints (drug resistance among treatment commencing participants, treatment failures, serious adverse events as detailed below) in accordance with a Statistical Analysis Plan under development before conclusion of recruitment. Access to identified study data will be limited to the data manager and study coordinator. Deidentified data will be limited to study investigators.

For peer review only - http://bmjopen.bmj.com/site/about/guidelines.xhtml

3.3 Adverse events

This study uses a drug with side effects that are already known. It will therefore report adverse events that, in the opinion of the investigator, are not deemed to be expected or are increased in severity from prior observations. Side effects not already known of the drug in a severity that the treating investigator deems as unexpected/serious should be reported to Burnet Institute as Sponsor.

For this study we will only be collecting adverse events that are inconsistent with prior observations reported in study drug product information. Adverse events considered to be related to study drug administration will be captured up until 24 weeks after the last recorded study dose.

Serious Adverse Events will be reported to the Burnet as Sponsor within 24 hours of the site learning of them and include any event that results in: Death; threat to life; significant disability or incapacity; congenital abnormality or birth defect; hospitalisation or is medically significant and requires intervention to prevent any of the former outcomes.

For peer review only

For peer review only - http://bmjopen.bmj.com/site/about/guidelines.xhtml

Burnet Institute is responsible for reporting safety events to the reviewing HREC in accordance with Safety Monitoring and Reporting in Clinical Trials Involving Therapeutic Goods (NHMRC, 2016).

In line with the Alfred Hospital Ethics Committee Safety Monitoring and Reporting Requirements (October 2017), only events that meet the criteria of a Significant Safety Issue and/or Urgent Safety Measure will need to be reported to the Alfred HREC.

3.4 Dissemination

Publication of data derived from the study will be supervised by the Protocol Steering Committee. The results of the project will be published and presented at scientific meetings. All published quantitative data will be non-identifiable grouped data. Proposals for additional projects or collaborations, likely to result in separate publications, will be reviewed by the Steering Committee for final determination. A plain English summary of study outcomes, as well as abstracts from publications, will be available on Burnet Institute website. The plain English summary of study outcomes will also be provided to all participating sites. Results may also form part of PhD or Honours student theses. Authorship for publications arising from

For peer review only - http://bmjopen.bmj.com/site/about/guidelines.xhtml

this study will adhere to the International Committee of Medical Journal Editors guidelines [41].

For peer review only

For peer review only - http://bmjopen.bmj.com/site/about/guidelines.xhtml

4 REFERENCES

1 World Health Organization. Hepatitis C: Fact Sheet. 2022.https://www.who.int/news-room/fact-sheets/detail/hepatitis-c (accessed 23 Jun 2022).

2 Burnet Institute. Australia's progress towards hepatitis C elimination: Annual report 2021. 2021. https://burnet.edu.au/system/asset/file/5001/BurnetKirby-hepC-2021-report.pdf

3 Grove A. The Pharmaceutical Benefits Scheme: a quick guide. 2018.https://www.aph.gov.au/About_Parliament/Parliamentary_Departments/Parliamentary_Library/pubs/rp/rp1516/Quick_Guides/PBS (accessed 15 Dec 2022).

4 Ghinea N, Lipworth W, Day R, *et al.* Importation of generic hepatitis C therapies: bridging the gap between price and access in high-income countries. *Lancet (London, England)* 2017;**389**:1268–72. doi:10.1016/S0140-6736(16)32051-7

5 Moon S, Erickson E. Universal Medicine Access through Lump-Sum Remuneration - Australia's Approach to Hepatitis C. *N Engl J Med* 2019;**380**:607–10. doi:10.1056/NEJMp1813728

6 Kirby Institute. HIV, viral hepatitis and sexually transmissible infections in Australia: annual surveillance report 2018. Sydney, Australia: 2018.

7 Scott N, McBryde ES, Thompson A, *et al.* Treatment scale-up to achieve global HCV incidence and mortality elimination targets: a cost-effectiveness model. *Gut* 2017;**66**:1507–15. doi:10.1136/gutjnl-2016-311504

8 Martin NK, Foster GR, Vilar J, *et al.* HCV treatment rates and sustained viral response among people who inject drugs in seven UK sites: real world results and modelling of treatment impact. *J Viral Hepat* 2015;**22**:399–408. doi:10.1111/jvh.12338

9 Wilkinson AL, van Santen DK, Traeger MW, *et al.* Hepatitis C incidence among patients attending primary care health services that specialise in the care of people who inject drugs, Victoria, Australia, 2009 to 2020. *Int J Drug Policy* 2022;**103**:103655.

For peer review only

doi:https://doi.org/10.1016/j.drugpo.2022.103655

10 Scott N, Doyle JS, Wilson DP, *et al.* Reaching hepatitis C virus elimination targets requires health system interventions to enhance care cascade. *Int J Drug Policy* 2017;**47**:107–16.

11 Scott N, Iser DM, Thompson AJ, *et al.* Cost-effectiveness of treating chronic hepatitis C virus with direct-acting antivirals in people who inject drugs in Australia. *J Gastroenterol Hepatol* 2016;**31**:872–82. doi:10.1111/jgh.13223

12 Sublette VA, Smith SK, George J, *et al.* HCV treatment experience: patients' perceptions of the facilitators of and barriers to uptake, adherence and compeletion. *Psychol Heal* 2015;**30**:987–1004.

13 Snow K, Scott N, Clothier HJ, *et al.* Limited provision of diagnostic services to Victorians living with HCV antibodies, 2001-2012: a multi-level modelling analysis. *Aust N Z J Public Health* 2016;**41**:193–8.

14 Gidding HF, Topp L, Middleton M, *et al.* The epidemiology of HCV in Australia: notifications, treatment uptake and liver transplantations. *J Gastroenterol Hepatol* 2009;**24**:1648–54.

15 Traeger MW, van Santen DK, Sacks-Davis R, *et al.* Impact of COVID-19 lockdown restrictions on hepatitis C testing in Australian primary care services providing care for people who inject drugs. *J Viral Hepat* 2022;**29**:908–18. doi:10.1111/jvh.13723

16 Doyle JS, Scott N, Sacks-Davis R, *et al.* Treatment access is only the first step to hepatitis C elimination. *Aliment Pharmacol Ther* 2019;**49**:1223–9.

17 Høj SB, Jacka B, Minoyan N, *et al.* Conceptualising access in the direct-acting antiviral era: An integrated framework to inform research and practice in HCV care for people who inject drugs. *Int J Drug Policy* 2019;**72**:11–23. doi:10.1016/j.drugpo.2019.04.001

18 Pourmarzi D, Smirnov A, Hall L, *et al.* Enablers and barriers for the provision of community-based HCV treatment: A case study of a real-world practice. *J Viral Hepat* 2020;**27**:484–96. doi:10.1111/jvh.13259

For peer review only

19 Latham NH, Pedrana A, Doyle JS, *et al.* Community-based, point-of-care hepatitis C testing: perspectives and preferences of people who inject drugs. *J Viral Hepat* 2019;**26**:919–22. doi:10.1111/jvh.13087

20 Thompson AJ V. Australian recommendations for the management of hepatitis C virus infection: a consensus statement. *Med J Aust* 2016;**204**:268–72. doi:10.5694/mja16.00106

21 Iversen J, Grebely J, Catlett B, *et al.* Estimating the cascade of hepatitis C testing, care and treatment among people who inject drugs in Australia. *Int J Drug Policy* 2017;**47**:77–85. doi:10.1016/j.drugpo.2017.05.022

22 The Kirby Institute. Australian NSP survey national data report 2016-2020. 2021.

23 Khuroo MS, Khuroo NS, Khuroo MS. Diagnostic accuracy of point-of-care tests for hepatitis C virus infection: a systematic review and meta-analysis. *PLoS One* 2015;**10**:e0121450. doi:10.1371/journal.pone.0121450

24 Grebely J, Lamoury FMJ, Hajarizadeh B, *et al.* Evaluation of the Xpert HCV Viral Load point-of-care assay from venepuncture-collected and finger-stick capillary whole-blood samples: a cohort study. *lancet Gastroenterol Hepatol* 2017;**2**:514–20. doi:10.1016/S2468-1253(17)30075-4

25 Williams B, Howell J, Doyle J, *et al.* Point-of-care hepatitis C testing from needle and syringe programs: An Australian feasibility study. *Int J Drug Policy* 2019;**72**:91–8. doi:10.1016/j.drugpo.2019.05.012

26 Jewett A, Al-Tayyib AA, Ginnett L, *et al.* Successful Integration of Hepatitis C Virus Point-of-Care Tests into the Denver Metro Health Clinic. *AIDS Res Treat* 2013;**2013**:528904. doi:10.1155/2013/528904

27 Khalid GG, Kyaw KWY, Bousquet C, *et al.* From risk to care: the hepatitis C screening and diagnostic cascade in a primary health care clinic in Karachi, Pakistan-a cohort study. *Int Health* 2018.

28 Bottero J, Boyd A, Gozlan J, *et al.* Simultaneous Human Immunodeficiency Virus-

For peer review only

For peer review only - http://bmjopen.bmj.com/site/about/guidelines.xhtml

Hepatitis B-Hepatitis C Point-of-Care Tests Improve Outcomes in Linkage-to-Care: Results of a Randomized Control Trial in Persons Without Healthcare Coverage. *Open forum Infect Dis* 2015;**2**:ofv162. doi:10.1093/ofid/ofv162

29 Howell J, Traeger MW, Williams B, *et al.* The impact of point-of-care hepatitis C testing in needle and syringe exchange programs on linkage to care and treatment uptake among people who inject drugs: An Australian pilot study. *J Viral Hepat* 2022;**29**:375–84. doi:10.1111/jvh.13664

30 Morano JP, Zelenev A, Lombard A, *et al.* Strategies for hepatitis C testing and linkage to care for vulnerable populations: point-of-care and standard HCV testing in a mobile medical clinic. *J Community Health* 2014;**39**:922–34. doi:10.1007/s10900-014-9932-9

31 Shiha G, Soliman R, Serwah A, *et al.* A same day ‘test and treat’ model for chronic HCV and HBV infection: Results from two community-based pilot studies in Egypt. *J Viral Hepat* 2020;**27**:593–601. doi:10.1111/jvh.13268

32 Eckhardt B, Kapadia SN, Mateu-Gelabert P, *et al.* Rapid Treatment Initiation for Hepatitis C in Young People Who Inject Drugs: The Seek, Test, and Rapid Treatment Randomized Trial. *Open Forum Infect Dis* 2022;**9**:ofac225. doi:10.1093/ofid/ofac225

33 Yoshida EM, Sulkowski MS, Gane EJ, *et al.* Concordance of sustained virological response 4, 12, and 24 weeks post-treatment with sofosbuvir-containing regimens for hepatitis C virus. *Hepatology* 2015;**61**:41–5. doi:10.1002/hep.27366

34 World Health Organization. Periodic presumptive treatment for sexually trasmitted infections. 2008.

35 Therapeutic Goods Administration. Australian Public Assessment Report for: Sofosbuvir/Velaptasvir. 2017.

36 The Shiny CRT Calculator: Power amd Sample size for Cluster Randomised Trials. https://clusterrcts.shinyapps.io/rshinyapp/ (accessed 5 Mar 2022).

37 Hemming K, Kasza J, Hooper R, *et al.* A tutorial on sample size calculation for multiple-period cluster randomized parallel, cross-over and stepped-wedge trials using the Shiny

For peer review only

For peer review only - http://bmjopen.bmj.com/site/about/guidelines.xhtml

CRT Calculator. *Int J Epidemiol* Published Online First: 22 February 2020. doi:10.1093/ije/dyz237

38 Campbell MK, Piaggio G, Elbourne DR, *et al.* Consort 2010 statement: extension to cluster randomised trials. *BMJ* 2012;**345**:e5661. doi:10.1136/bmj.e5661

39 Morgan KE, Forbes AB, Keogh RH, *et al.* Choosing appropriate analysis methods for cluster randomised cross-over trials with a binary outcome. *Stat Med* 2017;**36**:318–33. doi:10.1002/sim.7137

40 Kenward MG, Roger JH. Small sample inference for fixed effects from restricted maximum likelihood. *Biometrics* 1997;**53**:983–97.

41 International Committee of Medical Journal Editors. Defining the role of authors and contributors: International Committee of Medical Journal Editors. 2019.

For peer review only

For peer review only - http://bmjopen.bmj.com/site/about/guidelines.xhtml

AUTHORS' CONTRIBUTIONS:

- KH and JD contributed equally to this paper
- JD and AEP conceived the project, have overall responsibility for the trial design and trial protocol, and reviewed the manuscript
- KH contributed to drafting of the trial protocol, contributed to the trial design and drafted the manuscript.
- CD, AW, IE, KA, SVB, RG, KC and BC contributed to the trial design and trial protocol and reviewed and edited the manuscript
- All authors provided critical review on the trial protocol and approved the final manuscript

FUNDING STATEMENT:

QuickStart is support by the National Health and Medical Reseach Council (GNT1188026) and an investigator-initiated grant from Gilead Sciences. The funders had no role in the conduct of the trial or decision to publish. The Burnet Institute receives support from the Victorian Government Operational Infrastructure Fund, unrelated to this work.

For peer review only

For peer review only - http://bmjopen.bmj.com/site/about/guidelines.xhtml

COMPETING INTERESTS STATEMENT:

JSD, MH, AEP report investigator-iniated funding to their institution from Gilead Sciences, AbbVie and Merck. JSD reports honoraria to his institution from Gilead Sciences and AbbVie.

For peer review only

SPIRIT

STANDARD PROTOCOL ITEMS: RECOMMENDATIONS FOR INTERVENTIONAL TRIALS

SPIRIT 2013 Checklist: Recommended items to address in a clinical trial protocol and related documents*

Section/item	Item No	Description	Page no
**Administrative information**			
Title	1	Descriptive title identifying the study design, population, interventions, and, if applicable, trial acronym	p1
Trial registration	2a	Trial identifier and registry name. If not yet registered, name of intended registry	p2
	2b	All items from the World Health Organization Trial Registration Data Set	n/a
Protocol version	3	Date and version identifier	p7
Funding	4	Sources and types of financial, material, and other support	p30
Roles and responsibilities	5a	Names, affiliations, and roles of protocol contributors	p1,p30
	5b	Name and contact information for the trial sponsor	p1
	5c	Role of study sponsor and funders, if any, in study design; collection, management, analysis, and interpretation of data; writing of the report; and the decision to submit the report for publication, including whether they will have ultimate authority over any of these activities	p30
	5d	Composition, roles, and responsibilities of the coordinating centre, steering committee, endpoint adjudication committee, data management team, and other individuals or groups overseeing the trial, if applicable (see Item 21a for data monitoring committee)	pp18-20
**Introduction**			
Background and rationale	6a	Description of research question and justification for undertaking the trial, including summary of relevant studies (published and unpublished) examining benefits and harms for each intervention	pp4-6
	6b	Explanation for choice of comparators	pp4-6
Objectives	7	Specific objectives or hypotheses	p6

For peer review only

Trial design	8	Description of trial design including type of trial (eg, parallel group, crossover, factorial, single group), allocation ratio, and framework (eg, superiority, equivalence, noninferiority, exploratory)	p7
**Methods: Participants, interventions, and outcomes**			
Study setting	9	Description of study settings (eg, community clinic, academic hospital) and list of countries where data will be collected. Reference to where list of study sites can be obtained	p7
Eligibility criteria	10	Inclusion and exclusion criteria for participants. If applicable, eligibility criteria for study centres and individuals who will perform the interventions (eg, surgeons, psychotherapists)	p8
Interventions	11a	Interventions for each group with sufficient detail to allow replication, including how and when they will be administered	pp8-11
	11b	Criteria for discontinuing or modifying allocated interventions for a given trial participant (eg, drug dose change in response to harms, participant request, or improving/worsening disease)	pp8-11
	11c	Strategies to improve adherence to intervention protocols, and any procedures for monitoring adherence (eg, drug tablet return, laboratory tests)	p17
	11d	Relevant concomitant care and interventions that are permitted or prohibited during the trial	n/a
Outcomes	12	Primary, secondary, and other outcomes, including the specific measurement variable (eg, systolic blood pressure), analysis metric (eg, change from baseline, final value, time to event), method of aggregation (eg, median, proportion), and time point for each outcome. Explanation of the clinical relevance of chosen efficacy and harm outcomes is strongly recommended	p18
Participant timeline	13	Time schedule of enrolment, interventions (including any run-ins and washouts), assessments, and visits for participants. A schematic diagram is highly recommended (see Figure)	pp15-17
Sample size	14	Estimated number of participants needed to achieve study objectives and how it was determined, including clinical and statistical assumptions supporting any sample size calculations	p18

Recruitment	15	Strategies for achieving adequate participant enrolment to reach target sample size	p7
**Methods: Assignment of interventions (for controlled trials)**			
Allocation:			
Sequence generation	16a	Method of generating the allocation sequence (eg, computer-generated random numbers), and list of any factors for stratification. To reduce predictability of a random sequence, details of any planned restriction (eg, blocking) should be provided in a separate document that is unavailable to those who enrol participants or assign interventions	p19
Allocation concealment mechanism	16b	Mechanism of implementing the allocation sequence (eg, central telephone; sequentially numbered, opaque, sealed envelopes), describing any steps to conceal the sequence until interventions are assigned	p19
Implementation	16c	Who will generate the allocation sequence, who will enrol participants, and who will assign participants to interventions	p19
Blinding (masking)	17a	Who will be blinded after assignment to interventions (eg, trial participants, care providers, outcome assessors, data analysts), and how	n/a
	17b	If blinded, circumstances under which unblinding is permissible, and procedure for revealing a participant's allocated intervention during the trial	n/a
**Methods: Data collection, management, and analysis**			
Data collection methods	18a	Plans for assessment and collection of outcome, baseline, and other trial data, including any related processes to promote data quality (eg, duplicate measurements, training of assessors) and a description of study instruments (eg, questionnaires, laboratory tests) along with their reliability and validity, if known. Reference to where data collection forms can be found, if not in the protocol	p19
	18b	Plans to promote participant retention and complete follow-up, including list of any outcome data to be collected for participants who discontinue or deviate from intervention protocols	p19

For peer review only - http://bmjopen.bmj.com/site/about/guidelines.xhtml

Data management	19	Plans for data entry, coding, security, and storage, including any related processes to promote data quality (eg, double data entry; range checks for data values). Reference to where details of data management procedures can be found, if not in the protocol	n/a
Statistical methods	20a	Statistical methods for analysing primary and secondary outcomes. Reference to where other details of the statistical analysis plan can be found, if not in the protocol	pp18-20
	20b	Methods for any additional analyses (eg, subgroup and adjusted analyses)	pp18-20
	20c	Definition of analysis population relating to protocol non-adherence (eg, as randomised analysis), and any statistical methods to handle missing data (eg, multiple imputation)	pp18-20
**Methods: Monitoring**			
Data monitoring	21a	Composition of data monitoring committee (DMC); summary of its role and reporting structure; statement of whether it is independent from the sponsor and competing interests; and reference to where further details about its charter can be found, if not in the protocol. Alternatively, an explanation of why a DMC is not needed	p21
	21b	Description of any interim analyses and stopping guidelines, including who will have access to these interim results and make the final decision to terminate the trial	n/a
Harms	22	Plans for collecting, assessing, reporting, and managing solicited and spontaneously reported adverse events and other unintended effects of trial interventions or trial conduct	pp21-22
Auditing	23	Frequency and procedures for auditing trial conduct, if any, and whether the process will be independent from investigators and the sponsor	p22
**Ethics and dissemination**			
Research ethics approval	24	Plans for seeking research ethics committee/institutional review board (REC/IRB) approval	p21

For peer review only - http://bmjopen.bmj.com/site/about/guidelines.xhtml

Protocol amendments	25	Plans for communicating important protocol modifications (eg, changes to eligibility criteria, outcomes, analyses) to relevant parties (eg, investigators, REC/IRBs, trial participants, trial registries, journals, regulators)	n/a
Consent or assent	26a	Who will obtain informed consent or assent from potential trial participants or authorised surrogates, and how (see Item 32)	p10, p22
	26b	Additional consent provisions for collection and use of participant data and biological specimens in ancillary studies, if applicable	n/a
Confidentiality	27	How personal information about potential and enrolled participants will be collected, shared, and maintained in order to protect confidentiality before, during, and after the trial	p22
Declaration of interests	28	Financial and other competing interests for principal investigators for the overall trial and each study site	p30
Access to data	29	Statement of who will have access to the final trial dataset, and disclosure of contractual agreements that limit such access for investigators	p21
Ancillary and post-trial care	30	Provisions, if any, for ancillary and post-trial care, and for compensation to those who suffer harm from trial participation	n/a
Dissemination policy	31a	Plans for investigators and sponsor to communicate trial results to participants, healthcare professionals, the public, and other relevant groups (eg, via publication, reporting in results databases, or other data sharing arrangements), including any publication restrictions	p22
	31b	Authorship eligibility guidelines and any intended use of professional writers	n/a
	31c	Plans, if any, for granting public access to the full protocol, participant-level dataset, and statistical code	p21
**Appendices**			
Informed consent materials	32	Model consent form and other related documentation given to participants and authorised surrogates	Appendix 1
Biological specimens	33	Plans for collection, laboratory evaluation, and storage of biological specimens for genetic or molecular analysis in the current trial and for future use in ancillary studies, if applicable	n/a

For peer review only - http://bmjopen.bmj.com/site/about/guidelines.xhtml

*It is strongly recommended that this checklist be read in conjunction with the SPIRIT 2013 Explanation & Elaboration for important clarification on the items. Amendments to the protocol should be tracked and dated. The SPIRIT checklist is copyrighted by the SPIRIT Group under the Creative Commons "Attribution-NonCommercial-NoDerivs 3.0 Unported" license.

For peer review only

For peer review only - http://bmjopen.bmj.com/site/about/guidelines.xhtml

BMJ Open

Same-visit hepatitis C testing and treatment to accelerate cure among people who inject drugs (The QuickStart Study): a cluster randomised crossover trial protocol

Journal:	*BMJ Open*
Manuscript ID	bmjopen-2023-083502.R1
Article Type:	Protocol
Date Submitted by the Author:	26-Mar-2024
Complete List of Authors:	Doyle, Joseph; Monash University, Infectious Diseases; Burnet Institute, Disease Elimination<br>Heath, Katherine; Burnet Institute<br>Elsum, Imogen; Burnet Institute<br>Douglass, Caitlin; Burnet Institute<br>Wade, Amanda; Burnet Institute<br>Kasza, Jessica; Monash University<br>Allardice, Kate; Burnet Institute, Disease Elimination<br>Von Bibra, Sally; Burnet Institute<br>Chan, Kico; Burnet Institute<br>Camesella, Beatriz; Burnet Institute<br>Guzman, Rodney; Burnet Institute<br>Bryant, Mellissa; Burnet Institute<br>Thompson, Alexander; St Vincent's Hospital Melbourne Pty Ltd, Gastroenterology; The University of Melbourne St Vincent's Department of Medicine<br>Stoové, Mark; Burnet Institute<br>Snelling, Thomas; Telethon Kids Institute, Wesfarmers Centre of Vaccines and Infectious Diseases; The University of Sydney Faculty of Medicine and Health<br>Scott, Nick; Burnet Institute<br>Spelman, Tim ; Burnet Institute<br>Anderson, David; Burnet Institute<br>Richmond, Jacqui; Burnet Institute<br>Howell, Jess; Burnet Institute; St Vincent's Hospital Melbourne Pty Ltd<br>Andric, Nada; HepatitisWA<br>Dietze, Paul; Burnet Institute; Monash University<br>Higgs, Peter; Burnet Institute; La Trobe University, Public Health<br>Sacks-Davis, Rachel ; Burnet Institute<br>Forbes, Andrew; Monash University, Epidemiology and Preventive Medicine<br>Hellard, Margaret; Burnet Institute; Monash University, Epidemiology and Preventive Medicine<br>Pedrana, Alisa; Burnet Institute; Monash University, Epidemiology and Preventive Medicine
**Primary Subject Heading**:	Infectious diseases

Secondary Subject Heading:	Gastroenterology and hepatology
Keywords:	Hepatology < INTERNAL MEDICINE, Public health < INFECTIOUS DISEASES, INFECTIOUS DISEASES

SCHOLARONE™
Manuscripts

TITLE:

Same-visit hepatitis C testing and treatment to accelerate cure among people who inject drugs (The QuickStart Study): a cluster randomised crossover trial protocol

AUTHORS:

Joseph S. Doyle*[1,2], Katherine Heath*[2], Imogen Elsum[2], Caitlin Douglass[2], Amanda J. Wade[2], Jessica Kasza[1], Kate Allardice[2], Sally Von Bibra[2], Kico Chan[2], Beatriz Camesella[2], Rodney Guzman[2], Mellissa Bryant[2], Alexander J. Thompson[3], Mark Stoové[2], Thomas L Snelling[4,5], Nick Scott[2], Tim Spelman[2], David Anderson[2], Jacqui Richmond[2], Jessica Howell[1,3], Nada Andric[6], Paul M. Dietze[1,2,7], Peter Higgs[2,8], Rachel Sacks-Davis[2], Andrew Forbes[1], Margaret E. Hellard[1,2], Alisa E. Pedrana[1,2]

joint first author

INSTITUTIONAL AFFILIATIONS:

[1] Monash University, Melbourne, VIC, Australia

[2] Burnet Institute, Melbourne, VIC, Australia

[3] St Vincent's Hospital and the University of Melbourne, Melbourne, VIC, Australia

[4] Telethon Kids Institute, Perth, WA, Australia

[5] University of Sydney, Sydney, NSW, Australia

[6] Hepatitis WA, Perth, WA, Australia

[7] National Drug Research Institute, Curtin University, Melbourne, VIC, Australia

[8] La Trobe University, Melbourne, VIC, Australia

CORRESPONDING AUTHOR:

Prof Joseph S. Doyle

Professor of Infectious Diseases, Monash University, and Deputy Director, Disease Elimination Program, Burnet Institute

For peer review only - http://bmjopen.bmj.com/site/about/guidelines.xhtml

85 Commercial Road, Melbourne VIC 3004 Australia
Email: Joseph.doyle@burnet.edu.au
Telephone: +61 3 9076 5436

ABSTRACT:

Introduction: Despite universal access to government funded Direct Acting Antivirals (DAAs) in 2016, the rate of hepatitis C treatment uptake in Australia has declined substantially. Most hepatitis C is related to injecting drug use; reducing the hepatitis C burden amongst people who inject drugs (PWID) is therefore paramount to reach hepatitis C elimination targets. Increasing DAA uptake by PWID is important for interrupting transmission and reducing incidence, as well as reducing morbidity and mortality and improving quality of life of people who inject drugs and meeting Australia's hepatitis C elimination targets.

Methods and analysis: A cluster randomised crossover trial will be conducted with three intervention arms and a control arm. Arm A will receive rapid hepatitis C virus (HCV) antibody testing; Arm B will receive rapid HCV antibody and rapid ribonucleic acid (RNA) testing; Arm C will receive rapid HCV antibody testing and same-day treatment initiation for HCV antibody positive participants; the Control Arm will receive standard of care. The primary outcomes will be (a) the proportion of participants with HCV commencing treatment and (b) the proportion of participants with HCV achieving cure. Analyses will be conducted on an intention-to-treat basis with mixed-effects logistic regression models.

Ethics and dissemination: The study has been approved by the Alfred Ethics Committee (Number HREC/64731/Alfred-2020-217547). Each participant will provide written informed consent. Reportable adverse events will be reported to the reviewing ethics committee. The findings will be presented at scientific conferences and published in peer-reviewed journals.

For peer review only - http://bmjopen.bmj.com/site/about/guidelines.xhtml

Registration details: ClinicalTrials.gov Identifier: NCT05016609

Trial progression: The study commenced recruitment on the 9 March 2022 and is expected to complete recruitment in December 2023.

STRENGTHS AND LIMITATIONS OF THIS STUDY:

- The QuickStart Trial will recruit from a focused population, people with current or past history of injecting drugs, who are most at risk of hepatitis C.
- A cross-over clustered randomised control trial design will be used to increase study power by leveraging within-site comparisons.
- Four study conditions will be investigated to analyse three rapid point-of-care (POC) testing and treatment interventions, compared with a control arm. One arm investigates a rapid POC antibody test and same day treatment - an intervention that could be utilised in resource limited settings if found to be effective.
- Medical record review will be used in addition to in-person participant follow-up to monitor study outcomes and reduce loss-to-follow up.
- The participant recruitment strategy counteracts several biases present in observational studies, but participation is voluntary, so a risk of selection bias remains.

For peer review only - http://bmjopen.bmj.com/site/about/guidelines.xhtml

1. INTRODUCTION

As of 2019, approximately 58 million people worldwide were living with hepatitis C (HCV), causing around 290,000 deaths annually [1]. In Australia, approximately 118,000 people were living with HCV at the end of 2020 [2].

Direct-acting antiviral medications (DAAs) are highly effective (cure rates >90%), have minimal side-effects and require only 8-12 weeks of daily tablets. The Australia Pharmaceutical Benefits Scheme is a government program that subsidises medicines to improve affordability [3]. In 2016, the Pharmaceutical Benefits Scheme negotiated a fixed-price contract providing access to DAAs for every Australian living with hepatitis C, giving Australia a unique opportunity to eliminate HCV [4,5].

Most untreated HCV infections in Australia are among people with a history of injecting drug use [2,6]. Models of HCV epidemics where transmission is driven by injecting drug use suggest that treating people who inject drugs (PWID) and people with a history of injecting drugs will lead to substantial reductions in HCV incidence and liver-related morbidity and mortality [7–9]. Indeed, models have shown that treating as few as 6% of the estimated population of PWID in Australia annually for ten years would reduce new HCV infections by 80% and HCV prevalence in PWID by 10% [10,11]. However, in Australia, PWID remain under-diagnosed, under-treated and under-serviced in HCV care and healthcare more broadly [2,12–14].

Unrestricted access to DAAs following government subsidy in 2016 resulted in an initial spike in treatment uptake due to increased availability of the medication to those who had previously been unable to access it. However treatment numbers have since declined, exacerbated by the COVID-19 pandemic [2,15,16]. There exist service, provider and individual barriers to

For peer review only

For peer review only - http://bmjopen.bmj.com/site/about/guidelines.xhtml

accessing HCV care [17]. One service-related barrier among PWID is the current need for multiple venepunctures in order to be diagnosed with HCV in Australia, particularly as venous access can be challenging in PWID [18,19]. Current clinical guidelines and funding rules require specific assessments before treatment can be started [20] necessitating multiple clinic visits. Data from the Australian Needle and Syringe Program Survey estimates that among the 93,000 PWID in Australia, the majority (89%) had a lifetime history of HCV antibody testing [21]. However, individuals are gradually lost throughout the cascade of care. Of Australian Needle and Syringe Program Survey participants reporting testing positive to an HCV antibody test, only 32% reported accessing HCV treatment up to 2020 (prior to the COVID19 pandemic) [22].

Point-of-care (POC) HCV testing can simplify diagnosis of HCV in healthcare settings. The emergence of rapid POC testing for both HCV antibodies and RNA has made this feasible. The OraQuick® rapid HCV antibody test is self-contained and uses fingerstick capillary blood samples to provide results in around 20 minutes. The GeneXpert ® HCV Viral Load test detects HCV RNA using fingerstick capillary blood to provide results in around 60 minutes. These tests have been shown to be highly sensitive and specific, [23,24] and overcome many of the barriers to treatment associated with the conventional care model.

Studies suggest POC testing is acceptable among PWID and can be feasibly integrated into health services [19,25–27]. However, limited data have assessed the impact of POC testing on treatment uptake. One study in France found a larger proportion of individuals who received a POC test completed HCV screening and were linked to care compared to those with standard serological testing [28]. Provisional data in Australia found POC testing through needle and

For peer review only - http://bmjopen.bmj.com/site/about/guidelines.xhtml

syringe exchange programs reduced time to treatment [29]. In Connecticut, patients selecting POC testing were more likely to be linked to HCV treatment than those opting for standard laboratory testing [30]. These studies suggest that POC testing has a positive effect upon retention in HCV care [27,29]. Beyond rapid testing, the feasibility of same-day testing and treatment with DAAs has been demonstrated: preliminary studies have shown high levels of linkage to care following same-day testing and treatment [31]. Same-day testing and treatment has also been shown to achieve high cure rates compared to facilitated referral [32]. However, only a limited number of studies that have assessed the impact on retention in care of same-day treatment following POC testing for HCV [31].

From these existing data, there are broadly three rapid testing models of care being contemplated and deployed in real-world settings: firstly, POC antibody testing alone (corresponding to Arm A of QuickStart Trial, more fully defined below); secondly, combined POC antibody and RNA testing (Arm B); and thirdly, combined POC testing and same-day treatment (Arm C). While promising in pilot studies, none of these models have been tested formally in clinical trials. Clinical trial evidence is required to determine effectiveness of POC testing with or without treatment compared with the current standard of clinical care.

The QuickStart Trial will firstly determine the effectiveness of rapid POC testing in primary healthcare settings upon treatment uptake. We hypothesise that rapid antibody testing alone (Arm A), and rapid antibody followed by immediate RNA testing (Arm B), will increase treatment uptake compared with standard clinical care (the control arm). Secondly, QuickStart will determine the effectiveness of POC antibody testing followed by same-day treatment (Arm C) upon cure. We hypothesis that testing and same-day treatment will increase cure compared

For peer review only - http://bmjopen.bmj.com/site/about/guidelines.xhtml

with standard clinical care. Our secondary outcomes will also compare each of the three intervention models to each other for any incremental benefit on treatment uptake and cure.

For peer review only

For peer review only - http://bmjopen.bmj.com/site/about/guidelines.xhtml

2. METHODS AND ANALYSIS

2.1 Trial design

A clustered randomised crossover trial with six treatment sequences will evaluate the effects of rapid POC testing on retention of participants with HCV care and achievement of cure. Each site will have a standard of care control period randomly occurring before or after one of three intervention arms, allowing for intra-site comparison. Each site will have a standard of care control period randomly occurring before or after one of the three intervention arms, allowing for intra-site and inter-site comparisons to be combined in the estimation of treatment effects. The cross-over design has advantages in ensuring that any external changes that occur over time can be accounted for, which a simple before and after intervention comparison would not allow. External changes might occur at the clinic level, such as services and staff becoming more aware or skilled in offering hepatitis C testing, or they may occur at a system level, such as changes in access to testing, care or treatment. There will be a one-month washout period between arms at each study site. Participant recruitment began in March 2022 and is intended to continue for up to 24 months.

2.2 Patient and Public Involvement

We engaged the University of New South Wales to convene a Community Reference Panel to provide input and feedback into the design of the research and study procedures. The panel involved ten members from the study target audience who were involved in two separate consultations in 2021.

For peer review only

2.3 Study setting

The trial will be conducted at primary healthcare services in metropolitan and rural settings in Australia with:

- high caseloads of people who currently or previously injected drugs and are at risk of HCV infection;
- a General Practitioner and/or a Nurse Practitioner experienced in testing and treating HCV

2.4 Recruitment

Eligible clinics will be identified through consultation with government, community, clinical and research networks in viral hepatitis in Australia. Within clinics, potentially eligible participants will be approached by a clinician, nurse or community health worker. Participant-facing study promotion including posters and leaflets will be used at the discretion of participating clinics.

2.5 Participant eligibility

Study inclusion criteria for participants are as follows:

- current or former injecting drug use (i.e., injected drugs at least once)
- aged 18 years or over
- attending a participating primary healthcare for any reason

For peer review only - http://bmjopen.bmj.com/site/about/guidelines.xhtml

- no previous treatment with DAAs
- medicare eligible
- able to speak and understand English

Exclusion criteria are as follows:

- women known to be currently pregnant or who are breastfeeding
- individuals currently engaged in HCV treatment
- unable to provide informed consent
- tested for HCV in the past 3 months
- previous successful treatment with interferon-based therapy for HCV

2.6 Study arms

This study will implement three intervention arms and a control arm, as shown in Figure 1. Final recruitment, retention and outcome assessments will be presented in accordance with the CONSORT statement, including CONSORT diagrams. Arm A will implement a rapid POC antibody test in addition to standard of care (SOC) confirmatory RNA testing and bloods tests at the baseline visit. Arm B will implement a rapid POC antibody and POC RNA test in addition to SOC bloods tests at the baseline visit. Arm C will implement a rapid POC antibody test and same-day treatment with DAAs for antibody positive participants in addition to standard bloods tests. The control arm will implement the SOC in operation at each clinic.

For peer review only - http://bmjopen.bmj.com/site/about/guidelines.xhtml

All participants will be administered a clinical and behavioural questionnaire at their baseline visit. All arms are outlined in detail below.

2.6.1 Arm A: Rapid POC antibody + SOC

Baseline visit:

- Participants will receive an OraQuick® rapid HCV antibody test using a fingerstick specimen, according to the manufacturer's instructions, with the result documented at 20 minutes. The result will be provided to the participant as a preliminary result with laboratory testing on a venous sample required to confirm the POC test result. If the POC result is positive, blood samples will be collected for any confirmatory laboratory antibody and RNA testing and SOC pre-treatment screening. If the POC result is negative, confirmatory testing will be offered but the participant is given the option to decline.

Week 0-2 (confirmatory lab results):

- If the confirmatory laboratory HCV Antibody result is **negative**, the nurse will communicate this to the participant. The participant will complete the study here. Standard harm minimisation messaging and window periods for detection of HCV will be discussed with the participant.

- If the confirmatory laboratory HCV antibody result is **positive** and the RNA result is **negative**, the nurse will contact the participant by phone to inform them of their result - prior exposure to HCV, no current infection. The participant will complete the study here.

For peer review only - http://bmjopen.bmj.com/site/about/guidelines.xhtml

- If the confirmatory laboratory HCV antibody result is **positive** and the RNA result is **positive**, the nurse will contact the participant by phone to inform them of their result of current HCV infection and arrange a follow up appointment to discuss treatment as per the clinic usual standard of care.

Week 2-12 (HCV RNA positive):

- HCV RNA positive participants will be followed-up as per usual clinic SOC.

Week 12 (HCV RNA positive):

- The study nurse will review the medical records of consented participants to determine whether participants with HCV were provided with a DAA prescription within 12 weeks of enrolment in the study.
- All participants who have been provided with a DAA prescription will be contacted by the study nurse to ask whether they started treatment and if so, when.

12 weeks following treatment completion (Sustained Virological Response (SVR) or cure assessment):

- The medical records of all participants confirmed through medical record review to have started DAA treatment will be reviewed to determine if they have had a SVR blood test anytime from 4 -12 weeks following their treatment completion date [33]. Reinfection will be differentiated from treatment failure due to relapse by assessing (1) genotype or subtype, and (2) viral sequencing at a reference laboratory, whenever possible.

For peer review only

For peer review only - http://bmjopen.bmj.com/site/about/guidelines.xhtml

- Participants who have not had a SVR visit, will be contacted by the study nurse approximately 12 weeks following the scheduled treatment completion date (determined by the date they received their script and duration of treatment) to request that they attend an SVR visit.

2.6.2 Arm B: Rapid POC antibody + rapid POC RNA + SOC

Baseline visit:

- Participants will receive an OraQuick® rapid HCV antibody test using a fingerstick specimen, according to the manufacturer's instructions, with the result documented at 20 minutes. The result from the 20-minute time point will be provided to the participant as a preliminary result, with laboratory testing on a venous sample required to confirm. Blood samples will be collected for any confirmatory laboratory testing and standard of care pre-treatment screening.
- Participants with a **negative** OraQuick® result will be provided with this preliminary result and informed that they will be contacted within the next two weeks with any laboratory test results.
- Participants with a **positive** OraQuick® result will be offered POC RNA testing using the Cepheid GeneXpert™ HCV RNA fingerstick test. The study nurse will conduct POC RNA testing using a fingerstick blood sample according to the manufacturer's instructions, with results in ~60 minutes. The study nurse will determine with the participant how they would like to receive these results – via remaining on site or by

For peer review only - http://bmjopen.bmj.com/site/about/guidelines.xhtml

phone call. If the HCV POC RNA is **negative** the study nurse will inform the participant of their result, either in person or by telephone, and they will be contacted within the next 2 weeks with confirmatory laboratory test results.

- If the HCV POC RNA is **positive**, the study nurse will inform the participant of their result either in person or by telephone and they will be contacted within the next two weeks with confirmatory laboratory test results.

Week 0-2 (confirmatory lab results):

- If the confirmatory laboratory RNA results are **negative** the nurse will inform the participant and the participant will complete the study. Standard harm minimisation messaging and window periods for detection of HCV will be discussed with the participant.
- If the laboratory HCV RNA is **positive** the study nurse will contact the participant to provide their results of current HCV infection and discuss follow up and treatment according to the practice of the clinic.

Week 2-12 (HCV RNA positive):

- Participants with HCV will be followed-up as per usual clinic SOC.

Week 12 (HCV RNA positive):

- The study nurse will review the medical records of consented participants to determine whether participants with HCV were provided with a DAA prescription within 12 weeks of enrolment in the study.

For peer review only

For peer review only - http://bmjopen.bmj.com/site/about/guidelines.xhtml

- All participants who have been provided with a DAA prescription, will be contacted by the study nurse to ask whether they started treatment and if so, when.

12 weeks following treatment completion (SVR):

SVR will be assessed as previously described for Arm A.

2.6.3 Arm C: Same-visit rapid POC antibody + treatment commencement

Baseline visit:

- Participants will receive an OraQuick® rapid HCV antibody test using a fingerstick specimen, according to the manufacturer's instructions, with the result documented at 20 minutes. The result from the 20-minute time point will be provided to the participant as a preliminary result, with laboratory testing on a venous sample required to confirm. Blood samples will be collected for any confirmatory laboratory testing and standard of care pre-treatment screening.
- Participants with a **negative** OraQuick® result will be advised that the POC test result will be confirmed by standard pathology results and they will be contacted in 1-2 weeks with these results.
- Participants with a **positive** OraQuick® result will be provided with two weeks of Sofosbuvir/Velpatasvir (Epclusa) and will be instructed to begin treatment. In this sense, Arm C uses presumptive treatment, where 14 days of treatment will be dispensed prior to laboratory confirmed diagnosis [34]. Blood samples will be sent for confirmatory HCV antibody and RNA testing. Participants of childbearing potential

For peer review only

will be screened for possible pregnancy and if indicated, a urinary pregnancy test will be performed before dispensing Epclusa. If the pregnancy test is positive, they will not be provided with study drug and will be referred to standard of care. Medical records will be reviewed for potential drug-drug interactions with Epclusa, and participants will be asked about medications they are currently taking. Participants taking medications with red or orange drug-drug interactions will not be provided with study drug and will be referred to standard of care.

Week 1-2 (Confirmatory Results)

- Participants will be contacted with the results of their confirmatory laboratory blood tests. Participants with a **positive** confirmatory antibody and **negative** RNA test result will be contacted with this result of prior exposure, no current infection and instructed to discontinue treatment and dispose of any unused medications. They will complete the study here. Standard harm minimisation messaging and window periods for detection of HCV will be discussed with the participant.
- Participants with a **positive** confirmatory antibody and **positive** confirmatory RNA result will be contacted to arrange on-going treatment through the study, a further four weeks of sofosbuvir/velpatasvir will be dispensed. Further assessment maybe required based on the laboratory blood test results:
    - If the participant received an AST:Platelet Ratio Index score of < 1.0, they will be instructed to continue treatment as they were originally instructed.
    - If the participant received an AST:Platelet Ratio Index score of ≥1.0, they will be instructed to continue taking the DAAs and will be referred to a specialised.

For peer review only

For peer review only - http://bmjopen.bmj.com/site/about/guidelines.xhtml

- ○ Participants found to have renal impairment (eGFR<30 ml/min) or coinfected with chronic hepatitis B will be instructed to cease treatment and will be referred to a specialist.
- ○ Participants found to have Human Immunodeficiency Virus infection will be instructed to continue the DAAs and be referred to a specialist.

Following specialist assessment, the participant may be required to change medications or alter their treatment course, according to the specialist's recommendations.

Week 6 (HCV RNA positive):

- Participants on treatment will be asked to attend a follow-up appointment where they will be provided with a further four weeks of sofosbuvir/velpatasvir.

Week 10 (HCV RNA positive):

- Participants who have received 10 weeks of treatment will be contacted to arrange a follow up appointment to receive the final two weeks of treatment.

12 weeks following treatment completion (SVR):

SVR will be assessed as previously described for Arm A.

2.6.4 Control arm: standard of care testing and treatment

All participants will be tested, treated and followed up according to the usual practice of the clinic for delivery of test results and ongoing HCV treatment. The participants medical record will be reviewed at the study close to assess testing and treatment uptake.

For peer review only

2.7 Study medication

For Arms A, B and the Control, medications will be funded by the PBS and dispensed utilising standard pharmacy practices. The type of DAAs used to treat participants with HCV in these Arms will be at the discretion of the clinical service where the participant is enrolled. For Arm C, study medications will be stored and dispensed by participating study sites. The medication used in Arm C of this study is a 12-week course of oral combination therapy sofosbuvir/velpatasvir (Epclusa™).

Epclusa tablets contain 400mg of sofosbuvir and 100mg of velpatasvir, to be taken orally once daily. Epclusa is TGA approved with ARTG ID 266823 [35].

2.8 Assignment of interventions

Intervention arms will be assigned to sites using random number generation in a centralised system. Sites will be randomised in batches of six to ensure a balance of the number of sites in each arm over time. Sites will be informed of their allocation to each of the two consecutive arms separately. Due to the nature of the study design, it is not possible to blind clinics or participants during all phases of the intervention. All sites randomised to Arms A, B or C in the first period will know that they will be allocated to the Control Arm after the cross-over period, due to the nature of the study design. Sites allocated to the Control Arm in the first period will be blinded as to which Arm they will be randomised to, following the cross-over washout period (see Figure 1).

For peer review only

For peer review only - http://bmjopen.bmj.com/site/about/guidelines.xhtml

2.9 Study outcomes

The two primary outcomes of this trial include:

(1) The proportion of participants commencing HCV treatment in each arm. Commencement of HCV treatment is defined as receipt of a script for treatment in arms A, B and Control and as commencement of treatment with study drug in Arm C. The proportion commencing treatment in each intervention arm will be compared to the Control arm.

(2) The proportion of participants with HCV who start treatment achieving cure. Cure will be assessed by review of medical records for a clinic visit to assess SVR; if no visit has taken place, the study nurse will contact the participant to arrange one in all Arms except the Control.

Commencing treatment is an important and easily measurable outcome in clinical practice. The rationale for two primary outcomes is that Arm C itself involves providing treatment to everyone found to have HCV as part of its intervention.

Secondary outcomes of this study include:

(1) Time to treatment commencement, defined as number of days from first test to first dose of treatment.

(2) Case finding, defined as the proportion of people diagnosed with HCV among all those recruited

(3) Cost-effectiveness, defined as the incremental cost to find and commence treatment among one person living with HCV from a health system perspective

(4) Participant acceptability, based on qualitative evaluation subject to further sub-study design and ethical approval

For peer review only

For peer review only - http://bmjopen.bmj.com/site/about/guidelines.xhtml

2.10 Sample size

Comparisons of primary interest are comparing outcome 1 (Treatment commencement) in Control and Arm A; outcome 1 in Control and Arm B; and outcome 2 (HCV cure) in Control and Arm C. We consider sample size calculations with a minimum of 80% power and a significance level of 0.05/3 = 0.0167. Comparisons of Control vs A are expected to result in the smallest required sample sizes, so sample size calculations focus on this comparison. We use the Shiny CRT app [36] for sample size calculations, assuming that the t- distribution is used instead of the normal approximation (accounting for small numbers of clusters) [37].

We suppose that five sites are assigned to each of the six treatment sequences. We found that with 24 participants recruited in each cluster in each period, power will be greater than 80% to detect a change in proportions of participants starting HCV treatment of 30% to 50% where ten clusters are allocated to Arm A. Due to greater portion of participants expected to start treatment in Arm B (70%) and Arm C (90%), power >80% is achieved with eight clusters assigned to the sequences that involve Arms B or C (i.e. with 4 clusters randomised to each of the Arm B then Control; Control then Arm B; Arm C then Control; and Control then Arm C sequences). The proportion of participants expected to achieve cure in each arm are 10% among controls, 30% in Arm A, 50% in Arm B, and 70% in Arm C. Allowing for 20% loss to follow up of sites and of participants, 30 participants per site per period would need to be recruited. Given these results, we will instruct sites to aim for 40 participants per site per period to counteract instances where some sites are unable to achieve 30 participants per site per period;

For peer review only

For peer review only - http://bmjopen.bmj.com/site/about/guidelines.xhtml

some sites achieving 40 participants will balance sites which achieve <30. This would imply a maximum total of 2,400 participants would need to be recruited.

2.11 Statistical analysis

Results from the trial will be reported according to the CONSORT extension statement for cluster randomised trials [38], or a CONSORT extension statement for cluster randomised crossover trials if one is available prior to end of data collection. Clinic and patient demographics will be described by site and study period. Analyses will be conducted on an intention-to-treat basis, with patients analysed according to the randomised treatment of their cluster at the time of their inclusion in the study. All available binary outcomes will be analysed at the patient level using mixed-effects logistic regression models with random intercepts for clinic and clinic-period and fixed effects for interventions [39]. Odds ratios and 95% confidence intervals will be reported; in addition, we will calculate and report risk differences and 95% confidence intervals.

There are three main comparisons proposed, namely, treatment uptake outcome in Arm A versus control (comparison one) and Arm B versus control (comparison two), and HCV cure in Arm C versus control (comparison three). Incremental benefits of Arm B to Arm A, and Arm C to Arm B, and Arm C to Arm A on treatment uptake and HCV cure will be performed in sub-group analyses. Data from controls in each arm will be pooled and a single model fitted for each outcome.

For peer review only - http://bmjopen.bmj.com/site/about/guidelines.xhtml

428 Randomisation will occur in batches of six, performed by the coordinating centre, and
429 concealed from investigators and sites until after site governance approcal. Since
430 randomisation is occurring within batches of six services, fixed effects for batch will be
431 included in these models. The Kenward-Roger correction will be applied to adjust for the small
432 number of clinics [40]. Estimates of intra-cluster correlations (within-period and between-
433 period) will be reported. Time-to-event outcomes such as time to complete diagnosis will be
434 analysed using Cox proportional hazards models with shared-frailty terms for site and fixed
435 terms for treatment group and randomisation batch. There may be imbalances between sites
436 with respect to various clinic and patient-level characteristics, and secondary analyses will
437 adjust for these.

438 Sensitivity analyses to investigate the impact of different assumptions about missing data on
439 study conclusions will be performed. Initially, two scenarios will be considered: "best-worst"
440 and "worst-best". In the "best-worst" scenario, all patients with missing data in clinics during
441 periods of administration of Arms A, B or C will be assumed to have commenced HCV
442 treatment, and all patients in control periods will be assumed not to have commenced HCV
443 treatment. In the "worst-best" scenario, all patients with missing data in clinics during periods
444 of administration of Arms A, B or C will be assumed to not have commenced HCV treatment,
445 and all patients in control periods will be assumed to have commenced HCV treatment. Similar
446 scenarios for the SVR outcome will be considered. Should these analyses lead to different study
447 conclusions, further analyses including terms predictive of missingness will be conducted or
448 using multiply imputed datasets will be considered.

For peer review only - http://bmjopen.bmj.com/site/about/guidelines.xhtml

3 ETHICS AND DISSEMINATION

3.1 Ethical review

This study has been approved by the Alfred Ethics Committee (Number: HREC/64731/Alfred-2020-217547). Informed consent will be obtained from all participants prior to enrolment. Potential participants will be presented with detailed information about the study procedures, objectives, risks and benefits. Participants will be able to refuse or terminate participation at any time. Participants will be reimbursed $40 at their baseline visit and $80 for their SVR visit. Reimbursement will be by Coles/Myer voucher or by direct funds bank transfer (EFT/PayID).

3.2 Data monitoring

The trial will be monitored by an independent Data Safety and Monitoring Board with an independent statistician. There will be pre-defined periodic analysis to assess safety endpoints (drug resistance among treatment commencing participants, treatment failures, serious adverse events as detailed below) in accordance with a Statistical Analysis Plan under development before conclusion of recruitment. Access to identified study data will be limited to the data manager and study coordinator. Deidentified data will be limited to study investigators.

3.3 Adverse events

This study uses a drug with side effects that are already known. It will therefore report adverse events that, in the opinion of the investigator, are not deemed to be expected or are increased in severity from prior observations. Side effects not already known of the drug in a severity

For peer review only - http://bmjopen.bmj.com/site/about/guidelines.xhtml

that the treating investigator deems as unexpected/serious should be reported to Burnet Institute as Sponsor.

For this study we will only be collecting adverse events that are inconsistent with prior observations reported in study drug product information. Adverse events considered to be related to study drug administration will be captured up until 24 weeks after the last recorded study dose.

Serious Adverse Events will be reported to the Burnet as Sponsor within 24 hours of the site learning of them and include any event that results in: Death; threat to life; significant disability or incapacity; congenital abnormality or birth defect; hospitalisation or is medically significant and requires intervention to prevent any of the former outcomes.

Burnet Institute is responsible for reporting safety events to the reviewing HREC in accordance with Safety Monitoring and Reporting in Clinical Trials Involving Therapeutic Goods (NHMRC, 2016).

In line with the Alfred Hospital Ethics Committee Safety Monitoring and Reporting Requirements (October 2017), only events that meet the criteria of a Significant Safety Issue and/or Urgent Safety Measure will need to be reported to the Alfred HREC.

3.4 Dissemination

Publication of data derived from the study will be supervised by the Protocol Steering Committee. The results of the project will be published and presented at scientific meetings. All published quantitative data will be non-identifiable grouped data. Proposals for additional

For peer review only - http://bmjopen.bmj.com/site/about/guidelines.xhtml

projects or collaborations, likely to result in separate publications, will be reviewed by the Steering Committee for final determination. A plain English summary of study outcomes, as well as abstracts from publications, will be available on Burnet Institute website. The plain English summary of study outcomes will also be provided to all participating sites. Results may also form part of PhD or Honours student theses. Authorship for publications arising from this study will adhere to the International Committee of Medical Journal Editors guidelines [41].

For peer review only

For peer review only - http://bmjopen.bmj.com/site/about/guidelines.xhtml

4 REFERENCES

1 World Health Organization. Hepatitis C: Fact Sheet. 2022.https://www.who.int/news-room/fact-sheets/detail/hepatitis-c (accessed 23 Jun 2022).

2 Burnet Institute. Australia's progress towards hepatitis C elimination: Annual report 2021. 2021. https://burnet.edu.au/system/asset/file/5001/BurnetKirby-hepC-2021-report.pdf

3 Grove A. The Pharmaceutical Benefits Scheme: a quick guide. 2018.https://www.aph.gov.au/About_Parliament/Parliamentary_Departments/Parliamentary_Library/pubs/rp/rp1516/Quick_Guides/PBS (accessed 15 Dec 2022).

4 Ghinea N, Lipworth W, Day R, *et al.* Importation of generic hepatitis C therapies: bridging the gap between price and access in high-income countries. *Lancet (London, England)* 2017;**389**:1268–72. doi:10.1016/S0140-6736(16)32051-7

5 Moon S, Erickson E. Universal Medicine Access through Lump-Sum Remuneration - Australia's Approach to Hepatitis C. *N Engl J Med* 2019;**380**:607–10. doi:10.1056/NEJMp1813728

6 Kirby Institute. HIV, viral hepatitis and sexually transmissible infections in Australia: annual surveillance report 2018. Sydney, Australia: 2018.

7 Scott N, McBryde ES, Thompson A, *et al.* Treatment scale-up to achieve global HCV incidence and mortality elimination targets: a cost-effectiveness model. *Gut* 2017;**66**:1507–15. doi:10.1136/gutjnl-2016-311504

8 Martin NK, Foster GR, Vilar J, *et al.* HCV treatment rates and sustained viral response among people who inject drugs in seven UK sites: real world results and modelling of treatment impact. *J Viral Hepat* 2015;**22**:399–408. doi:10.1111/jvh.12338

9 Wilkinson AL, van Santen DK, Traeger MW, *et al.* Hepatitis C incidence among patients attending primary care health services that specialise in the care of people who inject drugs, Victoria, Australia, 2009 to 2020. *Int J Drug Policy* 2022;**103**:103655. doi:https://doi.org/10.1016/j.drugpo.2022.103655

10 Scott N, Doyle JS, Wilson DP, *et al.* Reaching hepatitis C virus elimination targets requires health system interventions to enhance care cascade. *Int J Drug Policy* 2017;**47**:107–16.

11 Scott N, Iser DM, Thompson AJ, *et al.* Cost-effectiveness of treating chronic hepatitis C virus with direct-acting antivirals in people who inject drugs in Australia. *J Gastroenterol Hepatol* 2016;**31**:872–82. doi:10.1111/jgh.13223

12 Sublette VA, Smith SK, George J, *et al.* HCV treatment experience: patients'

For peer review only - http://bmjopen.bmj.com/site/about/guidelines.xhtml

perceptions of the facilitators of and barriers to uptake, adherence and compeletion. *Psychol Heal* 2015;**30**:987–1004.

13 Snow K, Scott N, Clothier HJ, *et al.* Limited provision of diagnostic services to Victorians living with HCV antibodies, 2001-2012: a multi-level modelling analysis. *Aust N Z J Public Health* 2016;**41**:193–8.

14 Gidding HF, Topp L, Middleton M, *et al.* The epidemiology of HCV in Australia: notifications, treatment uptake and liver transplantations. *J Gastroenterol Hepatol* 2009;**24**:1648–54.

15 Traeger MW, van Santen DK, Sacks-Davis R, *et al.* Impact of COVID-19 lockdown restrictions on hepatitis C testing in Australian primary care services providing care for people who inject drugs. *J Viral Hepat* 2022;**29**:908–18. doi:10.1111/jvh.13723

16 Doyle JS, Scott N, Sacks-Davis R, *et al.* Treatment access is only the first step to hepatitis C elimination. *Aliment Pharmacol Ther* 2019;**49**:1223–9.

17 Høj SB, Jacka B, Minoyan N, *et al.* Conceptualising access in the direct-acting antiviral era: An integrated framework to inform research and practice in HCV care for people who inject drugs. *Int J Drug Policy* 2019;**72**:11–23. doi:10.1016/j.drugpo.2019.04.001

18 Pourmarzi D, Smirnov A, Hall L, *et al.* Enablers and barriers for the provision of community-based HCV treatment: A case study of a real-world practice. *J Viral Hepat* 2020;**27**:484–96. doi:10.1111/jvh.13259

19 Latham NH, Pedrana A, Doyle JS, *et al.* Community-based, point-of-care hepatitis C testing: perspectives and preferences of people who inject drugs. *J Viral Hepat* 2019;**26**:919–22. doi:10.1111/jvh.13087

20 Thompson AJ V. Australian recommendations for the management of hepatitis C virus infection: a consensus statement. *Med J Aust* 2016;**204**:268–72. doi:10.5694/mja16.00106

21 Iversen J, Grebely J, Catlett B, *et al.* Estimating the cascade of hepatitis C testing, care and treatment among people who inject drugs in Australia. *Int J Drug Policy* 2017;**47**:77–85. doi:10.1016/j.drugpo.2017.05.022

22 The Kirby Institute. Australian NSP survey national data report 2016-2020. 2021.

23 Khuroo MS, Khuroo NS, Khuroo MS. Diagnostic accuracy of point-of-care tests for hepatitis C virus infection: a systematic review and meta-analysis. *PLoS One* 2015;**10**:e0121450. doi:10.1371/journal.pone.0121450

24 Grebely J, Lamoury FMJ, Hajarizadeh B, *et al.* Evaluation of the Xpert HCV Viral Load point-of-care assay from venepuncture-collected and finger-stick capillary whole-blood samples: a cohort study. *lancet Gastroenterol Hepatol* 2017;**2**:514–20.

For peer review only

doi:10.1016/S2468-1253(17)30075-4

25 Williams B, Howell J, Doyle J, *et al.* Point-of-care hepatitis C testing from needle and syringe programs: An Australian feasibility study. *Int J Drug Policy* 2019;**72**:91–8. doi:10.1016/j.drugpo.2019.05.012

26 Jewett A, Al-Tayyib AA, Ginnett L, *et al.* Successful Integration of Hepatitis C Virus Point-of-Care Tests into the Denver Metro Health Clinic. *AIDS Res Treat* 2013;**2013**:528904. doi:10.1155/2013/528904

27 Khalid GG, Kyaw KWY, Bousquet C, *et al.* From risk to care: the hepatitis C screening and diagnostic cascade in a primary health care clinic in Karachi, Pakistan-a cohort study. *Int Health* 2018.

28 Bottero J, Boyd A, Gozlan J, *et al.* Simultaneous Human Immunodeficiency Virus-Hepatitis B-Hepatitis C Point-of-Care Tests Improve Outcomes in Linkage-to-Care: Results of a Randomized Control Trial in Persons Without Healthcare Coverage. *Open forum Infect Dis* 2015;**2**:ofv162. doi:10.1093/ofid/ofv162

29 Howell J, Traeger MW, Williams B, *et al.* The impact of point-of-care hepatitis C testing in needle and syringe exchange programs on linkage to care and treatment uptake among people who inject drugs: An Australian pilot study. *J Viral Hepat* 2022;**29**:375–84. doi:10.1111/jvh.13664

30 Morano JP, Zelenev A, Lombard A, *et al.* Strategies for hepatitis C testing and linkage to care for vulnerable populations: point-of-care and standard HCV testing in a mobile medical clinic. *J Community Health* 2014;**39**:922–34. doi:10.1007/s10900-014-9932-9

31 Shiha G, Soliman R, Serwah A, *et al.* A same day ‘test and treat’ model for chronic HCV and HBV infection: Results from two community-based pilot studies in Egypt. *J Viral Hepat* 2020;**27**:593–601. doi:10.1111/jvh.13268

32 Eckhardt B, Kapadia SN, Mateu-Gelabert P, *et al.* Rapid Treatment Initiation for Hepatitis C in Young People Who Inject Drugs: The Seek, Test, and Rapid Treatment Randomized Trial. *Open Forum Infect Dis* 2022;**9**:ofac225. doi:10.1093/ofid/ofac225

33 Yoshida EM, Sulkowski MS, Gane EJ, *et al.* Concordance of sustained virological response 4, 12, and 24 weeks post-treatment with sofosbuvir-containing regimens for hepatitis C virus. *Hepatology* 2015;**61**:41–5. doi:10.1002/hep.27366

34 World Health Organization. Periodic presumptive treatment for sexually trasmitted infections. 2008.

35 Therapeutic Goods Administration. Australian Public Assessment Report for: Sofosbuvir/Velaptasvir. 2017.

36 The Shiny CRT Calculator: Power amd Sample size for Cluster Randomised Trials.

For peer review only

For peer review only - http://bmjopen.bmj.com/site/about/guidelines.xhtml

https://clusterrcts.shinyapps.io/rshinyapp/ (accessed 5 Mar 2022).

37 Hemming K, Kasza J, Hooper R, *et al.* A tutorial on sample size calculation for multiple-period cluster randomized parallel, cross-over and stepped-wedge trials using the Shiny CRT Calculator. *Int J Epidemiol* Published Online First: 22 February 2020. doi:10.1093/ije/dyz237

38 Campbell MK, Piaggio G, Elbourne DR, *et al.* Consort 2010 statement: extension to cluster randomised trials. *BMJ* 2012;**345**:e5661. doi:10.1136/bmj.e5661

39 Morgan KE, Forbes AB, Keogh RH, *et al.* Choosing appropriate analysis methods for cluster randomised cross-over trials with a binary outcome. *Stat Med* 2017;**36**:318–33. doi:10.1002/sim.7137

40 Kenward MG, Roger JH. Small sample inference for fixed effects from restricted maximum likelihood. *Biometrics* 1997;**53**:983–97.

41 International Committee of Medical Journal Editors. Defining the role of authors and contributors: International Committee of Medical Journal Editors. 2019.

FIGURE LEGENDS

Figure 1A. Sequence of events in Arm A: rapid HCV antibody testing followed by standard of care.

Figure 1B. Sequence of events in Arm B: rapid HCV antibody +/- rapid HCV RNA followed by standard of care.

Figure 1C. Sequence of events in Arm C: rapid HCV antibody followed by same-day treatment with DAAs.

Figure 1D. Sequence of events in the Control arm: standard of care

AUTHORS' CONTRIBUTIONS:

- Conception: JSD, MEH, AEP

For peer review only

- Design and development: JSD, KH, IE, AJW, JK, KA, MB, AJT, TLS, TSpelman, PMD, PH, AF, MEH, AEP
- Conduct and acquisition of Data: JSD, KH, IE, CD, AJW, KA, SVB, KC, BC, RG, MB, JR, NA, PH, AEP
- Analysis and interpretation: JSD, KH, JK, AJT, MS, TLS, NS, TSpelman, DA, RSD, JH, AF, MEH, AEP
- Drafting, critical review and approval of manuscript: JSD, KH, IE, CD, AJW, JK, KA, SVB, KC, BC, RG, MB, AJT, MS, TLS, NS, TSpelman, DA, JR, JH, NA, PMD, PH, RSD, AF, MEH, AEP
- Guarantor: JSD, AEP (co-principal investigators)
- JSD and KH contributed equally to this manuscript

FUNDING STATEMENT:

QuickStart is support by the National Health and Medical Research Council (GNT1188026) and an investigator-initiated grant from Gilead Sciences (no grant number). The funders had no role in the conduct of the trial or decision to publish. The Burnet Institute receives support from the Victorian Government Operational Infrastructure Fund (no grant number), unrelated to this work.

COMPETING INTERESTS STATEMENT:

For peer review only - http://bmjopen.bmj.com/site/about/guidelines.xhtml

JSD, MH, AEP report investigator-iniated funding to their institution from Gilead Sciences, AbbVie and Merck. JSD reports honoraria to his institution from Gilead Sciences and AbbVie.

For peer review only

Figure 1A. Sequence of events in Arm A: rapid HCV antibody testing followed by standard of care.

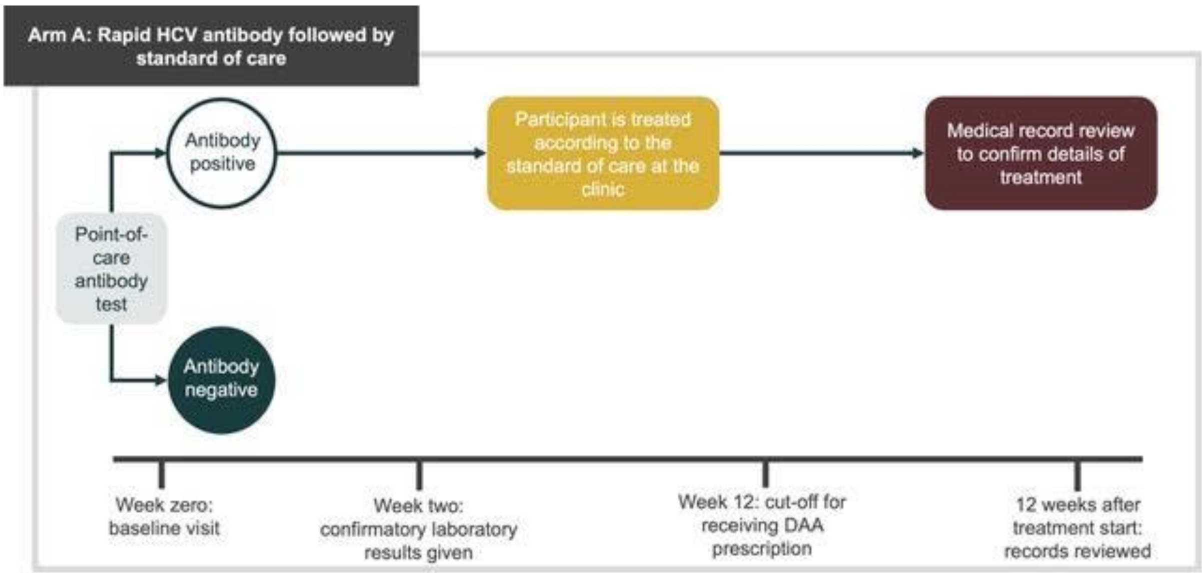

Figure 1B. Sequence of events in Arm B: rapid HCV antibody +/- rapid HCV RNA followed by standard of care.

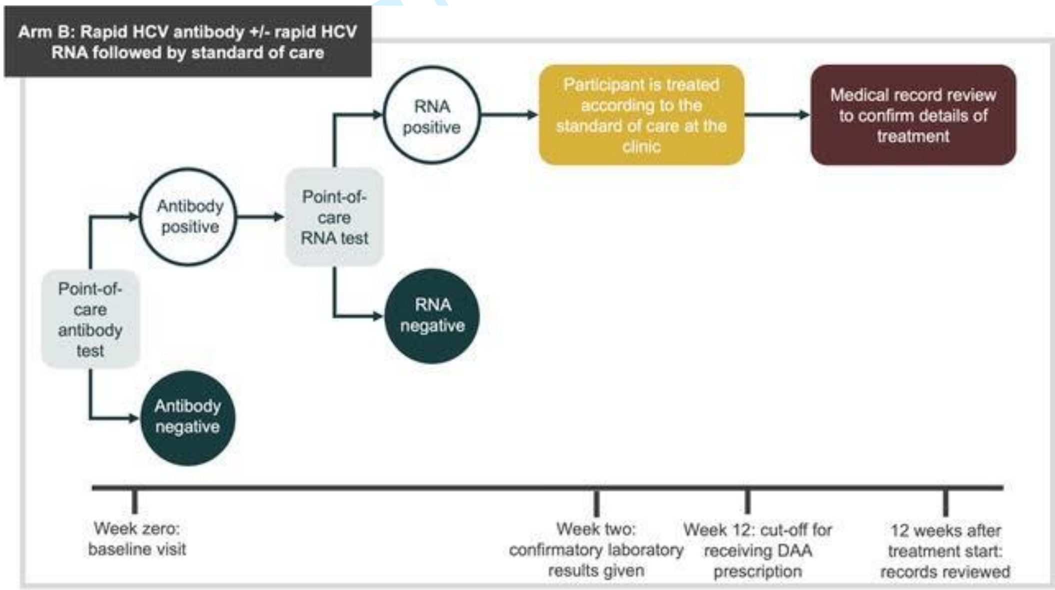

Figure 1C. Sequence of events in Arm C: rapid HCV antibody followed by same-day treatment with DAAs.

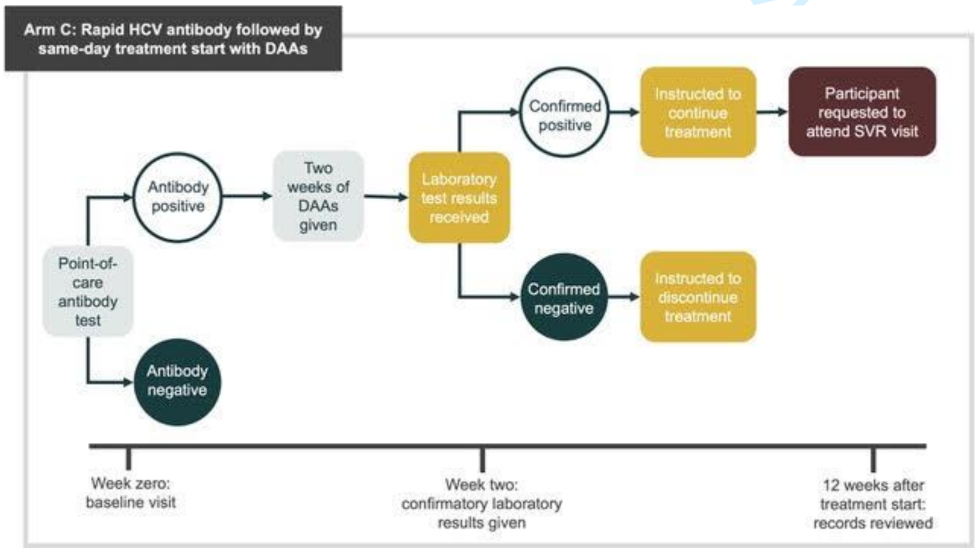

Figure 1D. Sequence of events in the Control arm: standard of care

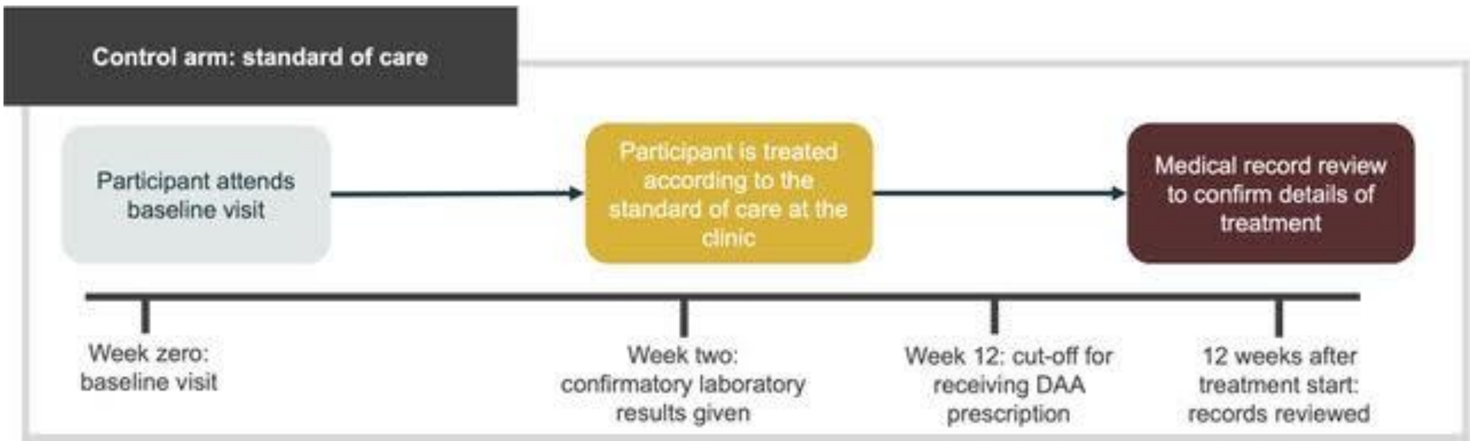

SPIRIT 2013 Checklist: Recommended items to address in a clinical trial protocol and related documents*

Section/item	Item No	Description	Page no
**Administrative information**			
Title	1	Descriptive title identifying the study design, population, interventions, and, if applicable, trial acronym	p1
Trial registration	2a	Trial identifier and registry name. If not yet registered, name of intended registry	p2
	2b	All items from the World Health Organization Trial Registration Data Set	n/a
Protocol version	3	Date and version identifier	p7
Funding	4	Sources and types of financial, material, and other support	p30
Roles and responsibilities	5a	Names, affiliations, and roles of protocol contributors	p1,p30
	5b	Name and contact information for the trial sponsor	p1
	5c	Role of study sponsor and funders, if any, in study design; collection, management, analysis, and interpretation of data; writing of the report; and the decision to submit the report for publication, including whether they will have ultimate authority over any of these activities	p30
	5d	Composition, roles, and responsibilities of the coordinating centre, steering committee, endpoint adjudication committee, data management team, and other individuals or groups overseeing the trial, if applicable (see Item 21a for data monitoring committee)	pp18-20
**Introduction**			
Background and rationale	6a	Description of research question and justification for undertaking the trial, including summary of relevant studies (published and unpublished) examining benefits and harms for each intervention	pp4-6
	6b	Explanation for choice of comparators	pp4-6
Objectives	7	Specific objectives or hypotheses	p6

For peer review only - http://bmjopen.bmj.com/site/about/guidelines.xhtml

Trial design	8	Description of trial design including type of trial (eg, parallel group, crossover, factorial, single group), allocation ratio, and framework (eg, superiority, equivalence, noninferiority, exploratory)	p7
**Methods: Participants, interventions, and outcomes**			
Study setting	9	Description of study settings (eg, community clinic, academic hospital) and list of countries where data will be collected. Reference to where list of study sites can be obtained	p7
Eligibility criteria	10	Inclusion and exclusion criteria for participants. If applicable, eligibility criteria for study centres and individuals who will perform the interventions (eg, surgeons, psychotherapists)	p8
Interventions	11a	Interventions for each group with sufficient detail to allow replication, including how and when they will be administered	pp8-11
	11b	Criteria for discontinuing or modifying allocated interventions for a given trial participant (eg, drug dose change in response to harms, participant request, or improving/worsening disease)	pp8-11
	11c	Strategies to improve adherence to intervention protocols, and any procedures for monitoring adherence (eg, drug tablet return, laboratory tests)	p17
	11d	Relevant concomitant care and interventions that are permitted or prohibited during the trial	n/a
Outcomes	12	Primary, secondary, and other outcomes, including the specific measurement variable (eg, systolic blood pressure), analysis metric (eg, change from baseline, final value, time to event), method of aggregation (eg, median, proportion), and time point for each outcome. Explanation of the clinical relevance of chosen efficacy and harm outcomes is strongly recommended	p18
Participant timeline	13	Time schedule of enrolment, interventions (including any run-ins and washouts), assessments, and visits for participants. A schematic diagram is highly recommended (see Figure)	pp15-17
Sample size	14	Estimated number of participants needed to achieve study objectives and how it was determined, including clinical and statistical assumptions supporting any sample size calculations	p18

For peer review only - http://bmjopen.bmj.com/site/about/guidelines.xhtml

Recruitment	15	Strategies for achieving adequate participant enrolment to reach target sample size	p7

Methods: Assignment of interventions (for controlled trials)

Allocation:

Sequence generation	16a	Method of generating the allocation sequence (eg, computer-generated random numbers), and list of any factors for stratification. To reduce predictability of a random sequence, details of any planned restriction (eg, blocking) should be provided in a separate document that is unavailable to those who enrol participants or assign interventions	p19
Allocation concealment mechanism	16b	Mechanism of implementing the allocation sequence (eg, central telephone; sequentially numbered, opaque, sealed envelopes), describing any steps to conceal the sequence until interventions are assigned	p19
Implementation	16c	Who will generate the allocation sequence, who will enrol participants, and who will assign participants to interventions	p19
Blinding (masking)	17a	Who will be blinded after assignment to interventions (eg, trial participants, care providers, outcome assessors, data analysts), and how	n/a
	17b	If blinded, circumstances under which unblinding is permissible, and procedure for revealing a participant's allocated intervention during the trial	n/a

Methods: Data collection, management, and analysis

Data collection methods	18a	Plans for assessment and collection of outcome, baseline, and other trial data, including any related processes to promote data quality (eg, duplicate measurements, training of assessors) and a description of study instruments (eg, questionnaires, laboratory tests) along with their reliability and validity, if known. Reference to where data collection forms can be found, if not in the protocol	p19
	18b	Plans to promote participant retention and complete follow-up, including list of any outcome data to be collected for participants who discontinue or deviate from intervention protocols	p19

Data management	19	Plans for data entry, coding, security, and storage, including any related processes to promote data quality (eg, double data entry; range checks for data values). Reference to where details of data management procedures can be found, if not in the protocol	n/a
Statistical methods	20a	Statistical methods for analysing primary and secondary outcomes. Reference to where other details of the statistical analysis plan can be found, if not in the protocol	pp18-20
	20b	Methods for any additional analyses (eg, subgroup and adjusted analyses)	pp18-20
	20c	Definition of analysis population relating to protocol non-adherence (eg, as randomised analysis), and any statistical methods to handle missing data (eg, multiple imputation)	pp18-20
**Methods: Monitoring**			
Data monitoring	21a	Composition of data monitoring committee (DMC); summary of its role and reporting structure; statement of whether it is independent from the sponsor and competing interests; and reference to where further details about its charter can be found, if not in the protocol. Alternatively, an explanation of why a DMC is not needed	p21
	21b	Description of any interim analyses and stopping guidelines, including who will have access to these interim results and make the final decision to terminate the trial	n/a
Harms	22	Plans for collecting, assessing, reporting, and managing solicited and spontaneously reported adverse events and other unintended effects of trial interventions or trial conduct	pp21-22
Auditing	23	Frequency and procedures for auditing trial conduct, if any, and whether the process will be independent from investigators and the sponsor	p22
**Ethics and dissemination**			
Research ethics approval	24	Plans for seeking research ethics committee/institutional review board (REC/IRB) approval	p21

For peer review only - http://bmjopen.bmj.com/site/about/guidelines.xhtml

Protocol amendments	25	Plans for communicating important protocol modifications (eg, changes to eligibility criteria, outcomes, analyses) to relevant parties (eg, investigators, REC/IRBs, trial participants, trial registries, journals, regulators)	n/a
Consent or assent	26a	Who will obtain informed consent or assent from potential trial participants or authorised surrogates, and how (see Item 32)	p10, p22
	26b	Additional consent provisions for collection and use of participant data and biological specimens in ancillary studies, if applicable	n/a
Confidentiality	27	How personal information about potential and enrolled participants will be collected, shared, and maintained in order to protect confidentiality before, during, and after the trial	p22
Declaration of interests	28	Financial and other competing interests for principal investigators for the overall trial and each study site	p30
Access to data	29	Statement of who will have access to the final trial dataset, and disclosure of contractual agreements that limit such access for investigators	p21
Ancillary and post-trial care	30	Provisions, if any, for ancillary and post-trial care, and for compensation to those who suffer harm from trial participation	n/a
Dissemination policy	31a	Plans for investigators and sponsor to communicate trial results to participants, healthcare professionals, the public, and other relevant groups (eg, via publication, reporting in results databases, or other data sharing arrangements), including any publication restrictions	p22
	31b	Authorship eligibility guidelines and any intended use of professional writers	n/a
	31c	Plans, if any, for granting public access to the full protocol, participant-level dataset, and statistical code	p21
**Appendices**			
Informed consent materials	32	Model consent form and other related documentation given to participants and authorised surrogates	Appendix 1
Biological specimens	33	Plans for collection, laboratory evaluation, and storage of biological specimens for genetic or molecular analysis in the current trial and for future use in ancillary studies, if applicable	n/a

For peer review only - http://bmjopen.bmj.com/site/about/guidelines.xhtml

*It is strongly recommended that this checklist be read in conjunction with the SPIRIT 2013 Explanation & Elaboration for important clarification on the items. Amendments to the protocol should be tracked and dated. The SPIRIT checklist is copyrighted by the SPIRIT Group under the Creative Commons "Attribution-NonCommercial-NoDerivs 3.0 Unported" license.

For peer review only
